# Variability of extracellular vesicle release during storage of red blood cell concentrates is associated with differential membrane alterations, including loss of cholesterol-enriched domains

**DOI:** 10.3389/fphys.2023.1205493

**Published:** 2023-06-20

**Authors:** Marine Ghodsi, Anne-Sophie Cloos, Negar Mozaheb, Patrick Van Der Smissen, Patrick Henriet, Christophe E. Pierreux, Nicolas Cellier, Marie-Paule Mingeot-Leclercq, Tomé Najdovski, Donatienne Tyteca

**Affiliations:** ^1^ Cell Biology Unit and Platform for Imaging Cells and Tissues, de Duve Institute, UCLouvain, Brussels, Belgium; ^2^ Cellular and Molecular Pharmacology Unit, Louvain Drug Research Institute, UCLouvain, Brussels, Belgium; ^3^ Service du Sang, Croix-Rouge de Belgique, Suarlée, Belgium

**Keywords:** red blood cell transfusion, intracellular ATP, oxidative stress, spectrin network, cholesterol, phosphatidylserine surface exposure, sphingomyelin-enriched domains, membrane microviscosity

## Abstract

Transfusion of red blood cell concentrates is the most common medical procedure to treat anaemia. However, their storage is associated with development of storage lesions, including the release of extracellular vesicles. These vesicles affect *in vivo* viability and functionality of transfused red blood cells and appear responsible for adverse post-transfusional complications. However, the biogenesis and release mechanisms are not fully understood. We here addressed this issue by comparing the kinetics and extents of extracellular vesicle release as well as red blood cell metabolic, oxidative and membrane alterations upon storage in 38 concentrates. We showed that extracellular vesicle abundance increased exponentially during storage. The 38 concentrates contained on average 7 × 10^12^ extracellular vesicles at 6 weeks (w) but displayed a ∼40-fold variability. These concentrates were subsequently classified into 3 cohorts based on their vesiculation rate. The variability in extracellular vesicle release was not associated with a differential red blood cell ATP content or with increased oxidative stress (in the form of reactive oxygen species, methaemoglobin and band3 integrity) but rather with red blood cell membrane modifications, i.e., cytoskeleton membrane occupancy, lateral heterogeneity in lipid domains and transversal asymmetry. Indeed, no changes were noticed in the low vesiculation group until 6w while the medium and the high vesiculation groups exhibited a decrease in spectrin membrane occupancy between 3 and 6w and an increase of sphingomyelin-enriched domain abundance from 5w and of phosphatidylserine surface exposure from 8w. Moreover, each vesiculation group showed a decrease of cholesterol-enriched domains associated with a cholesterol content increase in extracellular vesicles but at different storage time points. This observation suggested that cholesterol-enriched domains could represent a starting point for vesiculation. Altogether, our data reveal for the first time that the differential extent of extracellular vesicle release in red blood cell concentrates did not simply result from preparation method, storage conditions or technical issues but was linked to membrane alterations.

## Introduction

Blood conservation outside the bloodstream started a century ago with the discovery of citrate as anticoagulant and the addition of dextrose. From that time, scientists have been trying to improve continuously blood storage conditions and the transfusion efficacy. Nowadays, thanks to leukoreduction and additive solutions such as Saline-Adenine-Glucose-Mannitol (SAGM), red blood cell concentrates (RCCs) can be conserved for 42 days at 2°C–6°C. However, despite these considerable advances, the storage period is characterized by the appearance and the accumulation of detrimental changes in erythrocytes, collectively termed as ‘storage lesions’ (Garcia-Roa et al., 2017). Among those lesions, the irreversible loss of plasma membrane through the formation and the release of extracellular vesicles (EVs) is particularly problematic as they affect the *in vivo* viability and functionality of transfused red blood cells (RBCs) (Barshtein et al., 2016). In addition, EVs seem to be responsible for adverse post-transfusional complications such as thromboembolic and immunomodulatory events as suggested by *in vitro* studies (Rubin et al., 2012; Rubin et al., 2013). Therefore, it appears essential to carefully investigate EV biogenesis and release mechanisms in RCCs in order to improve RBC quality maintenance and to reduce risks of potential adverse effects upon transfusion.

Vesiculation seems to be the consequence of a series of lesions, supposed to be mainly induced by the development of oxidative stress. Throughout storage, the glycolysis pathway is reduced by the accumulation of lactate as well as the hypothermic and acidic storage conditions. As a result, high energy (ATP) and reducing (NAD(P)H and glutathione) compounds are progressively depleted. Moreover, due to the high concentration of ferrous ions and oxygen, chemical oxidation takes place. Since antioxidant defences are affected by metabolic impairments and the low temperature, they are rapidly overwhelmed, leading to the development of oxidative stress. The produced reactive oxygen species (ROS) then oxidize cytosolic and membrane proteins as well as lipids. This phenomenon has been proposed to (i) disturb the RBC cytoskeleton network and anchorage (Kriebardis et al., 2007), (ii) initiate the lipid peroxidation cycle (D'Alessandro et al., 2012), and (iii) disrupt the transversal membrane asymmetry by triggering the surface exposure of phosphatidylserine (PS), normally confined to the inner leaflet (Lu et al., 2011). In consequence, oxidative stress-related damage might finally lead to plasma membrane loss through vesiculation.

Nevertheless, literature data on vesiculation are difficult to compare due to differential RCC preparation and storage conditions (*e.g.*, different storage solutions or leukoreduction protocols) and varying experimental procedures to evidence the storage lesions. Additionally, we still do not properly understand (i) the precise succession of events, (ii) the exact link between the different parameters due to the labile boundary between cause and effect and (iii) if other mechanisms could be involved in EV biogenesis independently of the oxidative stress (Orlov and Karkouti, 2015; Yoshida et al., 2019).

Among the alternative mechanisms, one could propose the budding of EVs from specific regions of the plasma membrane such as submicrometric lipid domains. We previously showed the coexistence of 3 types of lipid domains at the outer plasma membrane leaflet of resting RBCs: (i) those mainly enriched in cholesterol (chol) (referred below as chol-enriched domains) and mostly located at the high curvature (HC) area of RBCs; (ii) those co-enriched in ganglioside GM1, phosphatidylcholine and chol (referred as GM1-enriched domains), found in the low curvature (LC) area, corresponding to the centre of the RBC; and (iii) those co-enriched in sphingomyelin (SM), phosphatidylcholine and chol (referred as SM-enriched domains), also found in LC areas (Carquin et al., 2014; Carquin et al., 2015; Conrard et al., 2018). During RBC deformation, GM1-enriched domain abundance increases in the LC area concomitantly with calcium influx while chol-enriched domains gather to increase the HC area needed for RBC deformation. After deformation, the SM-enriched domain number rises in parallel to calcium efflux in order to restore the initial discoid shape (Leonard et al., 2017b; Conrard et al., 2018; Leonard et al., 2018).

During the storage of RBCs in K^+^/EDTA-coated tubes at 4°C, we observed that, among the 3 types of lipid domains at the RBC area, only those enriched in chol are reduced in abundance (Cloos et al., 2020), suggesting a fine tuning of lipid domain dynamics during storage. This decrease occurs concomitantly with the increase of EV release and is preceded by a transient increase in the membrane microviscosity which could create a line tension between the bulk membrane and lipid domains and lead to their loss by vesiculation (Vind-Kezunovic et al., 2008; Leonard et al., 2018). Although storage of RBCs in K^+^/EDTA tubes accelerates and exacerbates the accumulation of lesions, this phenomenon could also occur during storage of RCCs as the chol-binding protein stomatin was found to decrease from RBC membranes and to be enriched in EVs during storage of RCCs (Salzer et al., 2008; Prudent et al., 2018).

The present study aimed at determining the mechanisms behind EV release in 38 RCCs by deciphering the time courses and extents of RBC metabolic (ATP concentration), oxidative (ROS and methaemoglobin (metHb) levels and band3 integrity) and membrane (membrane:cytoskeleton interactions, membrane microviscosity, lateral heterogeneity in lipid domains and transversal asymmetry) alterations. Based on data generated for those RBC parameters, we then evaluated the chol composition as well as the content of some membrane and cytoskeletal proteins in the isolated EVs.

## Materials and methods

### RBC concentrate preparation and whole blood collection

The study was approved by the Medical Ethics Committee of the Cliniques universitaires Saint-Luc (Brussels, Belgium). Leukoreduced RCCs were prepared by La Croix-Rouge de Belgique (Suarlée, Belgium) according to standard protocols defined by European legislations. Briefly, 450 ± 60 mL of whole blood were drawn by venipuncture from donor volunteers and collected into blood bags containing 63 mL of citrate-phosphate-dextrose solution (anticoagulant). Whole blood units were rapidly cooled and maintained at 18°C–22°C overnight. Next, RBCs were separated from plasma and buffy coat by centrifugation at 4,000 g for 10 min, resuspended in 100 mL of SAGM additive solution, leukoreduced by filtration and stored at 2°C–6°C. In total 38 RCCs from 36 different donors were included into the study. Seven RCCs entered the study immediately after preparation and were stored in our laboratory at 2°C–6°C for 2–70 days, while the 31 others were stored at La Croix-Rouge de Belgique for 2 weeks (w) (n = 3), 3w (n = 12), 4w (n = 5), 5w (n = 4) or 6w (n = 7) before delivery to our laboratory after ordering. All donors provided written consent for the use of their donation for scientific research. Donors were aged between 28 and 64 years and covered 20 men and 16 women. To avoid experimental variability and to determine a basal level for each parameter, a fresh blood tube from a female donor was selected as the internal and reference control for comparison with RCCs. However, as few is known about the *ex vivo* evolution of RBC membrane parameters, we ensured that this reference donor was not significantly different from additional donors (3 men and 5 women aged between 25–35 years). Fresh whole blood tubes were collected by venipuncture after informed consent and corresponded to citrate-coated blood tubes. K^+^/EDTA-coated tubes were also used for the measurement of lipid domain abundance (see below).

### RBC preparation

Except for EV isolation, metHb and extracellular K^+^ and glucose measurements, blood (from tubes) or RBCs (from RCCs) were diluted in the appropriate medium (10-fold and 13-fold, respectively) and washed before experiments, as decribed in (Cloos et al., 2020). Dulbecco’s Modified Eagle Medium (DMEM, Invitrogen) was employed for PS surface exposure, lipid domain abundance and membrane microviscosity measurement while Hanks’ Balanced Salt Solution (HBSS, Cytiva) without calcium was used for all other experiments.

### Particle/EV abundance, size and morphology

Particle/EV isolation was performed as in (Cloos et al., 2020). Briefly, RBCs were centrifuged 2 times at 2,000 g for 15 min at room temperature (RT) to collect the supernatant. Then, 800 µL of supernatant were diluted 8.75-fold in sterile filtered phosphate-buffered saline (PBS, Cytiva) solution and centrifuged one last time at 2,000 g at RT for 10 min. The resulting supernatant was submitted to ultracentrifugation at 20,000 g for 20 min at 4°C to pellet particles. After particle resuspension in sterile PBS, samples were submitted to a second ultracentrifugation step in the same conditions. The final pellet was resuspended in 1 mL of sterile PBS. Particle abundance and size were determined on freshly isolated particle samples by Nanoparticle Tracking Analysis (NTA) with the Zetaview (Particle Metrix). Samples were diluted 5- to 5,000-fold in sterile PBS depending on the initial sample concentration and determined for particle size (in nm) and abundance (in particles/mL). The particle concentration was converted into a particle number/RBC by considering the volume of supernatant engaged and the average number of RBCs/µL in RCCs.

Freshly isolated particles were prepared for electron microscopy as in (Cloos et al., 2020). Briefly, particles were immobilized on poly-L-Lysine (PLL)-coated coverslips at RT, washed with 0.1 M cacodylate, fixed with 1% glutaraldehyde in 0.1 M cacodylate, critical-point dried, sputter-coated with 10 nm of gold and finally observed in a CM12 electron microscope with SED detector at 80 kV.

### RBC morphology

RBCs were fixed in suspension for 15 min at RT in a solution containing 4% paraformaldehyde and 0.05% glutaraldehyde (v:v). Fixed RBCs were then washed and dropped off on PLL-coated coverslips for 8 min. Coverslips were finally mounted with Dako (Invitrogen) on SuperFrost Plus adhesion slides (VWR) and observed by light microscopy. RBCs were classified into discocytes, echinocytes and spherocytes. Their respective abundance was assessed through manual counting and expressed as % of the total RBC population.

### RBC ATP level

ATP levels were determined with the luminescent ATP detection assay kit (Abcam) as in (Cloos et al., 2020; Pollet et al., 2020). Data were normalised by the Hb content evaluated spectrophotometrically and expressed as % of fresh citrate-coated blood tubes.

### RBC reactive oxygen species and methaemoglobin levels

Intracellular ROS levels were detected by using the 2,7-dichlorodihydrofluoresceindiacetate (H_2_DCFDA, Invitrogen) probe as in (Cloos et al., 2020), except that the experiment was performed in calcium-free HBSS. The median fluorescence intensity (MFI) for RCCs was expressed as % of the MFI calculated for fresh citrate blood tubes. For positive control, 15 × 10^7^ RBCs from fresh blood tubes were treated with 10 µmol H_2_O_2_ for 3 min. Intracellular metHb levels were assessed with the automated blood gas analyser ABL-90 (Radiometer) from the Cliniques universitaires Saint-Luc (Brussels, Belgium). As the device detects metHb in whole blood, RBCs were not washed before measurement. The metHb amount was expressed as % of total Hb levels (oxygenated Hb, deoxygenated Hb, carboxyHb and metHb). The physiological range of metHb levels corresponded to the values provided in the ABL-90 manual. For positive control, 15 × 10^7^ RBCs from fresh blood tubes were treated with 3 µmol H_2_O_2_ for 15 min.

### RBC phosphatidylserine surface exposure

RBCs were labelled with Annexin-V-FITC (Invitrogen) and analysed by flow cytometry as in (Cloos et al., 2020). Since Annexin-V labelling requires the presence of calcium ions, calcium-containing DMEM was used for this experiment. The % of PS-exposing RBCs in fresh citrate-coated blood tubes was subtracted from the % of PS-exposing RBCs in concentrates.

### RBC spectrin immunofluorescence

Immunolabelling of α,β-spectrin was performed as in (Cloos et al., 2020; Pollet et al., 2020), except that RBCs were blocked with 3% bovine serum albumin (BSA, Sigma) in PBS for 60 min and that coverslips were mounted with Dako and examined with the Zeiss confocal microscope LSM980 using a plan-Apochromat 63x NA 1.4 oil immersion objective. The illumination settings used were identical for all samples from the same experiment. The membrane spectrin occupancy was determined with the Fiji software and data from RCCs were expressed as % of data obtained on fresh citrate-coated tubes.

### RBC cholesterol and sphingomyelin vital imaging

Chol and SM were visualized on living RBCs by fluorescence microscopy using respectively the mCherry-Theta toxin fragment and the fluorescent lipid analog BODIPY-SM (Invitrogen) as detailed in (Conrard et al., 2018; Cloos et al., 2020; Cloos et al., 2021). The mCherry-Theta toxin fragment concentrations ranged from 0.55 to 1.75 µM, due to several productions and purifications during the study. Lipid domain abundance was assessed through manual counting, normalised by the average hemi-RBC area calculated with the Fiji software and finally expressed as % of lipid domain abundance in fresh citrate-coated tubes or K^+^/EDTA-coated tubes (usually used in our laboratory for lipid domain imaging). While the abundance of SM-enriched domains was similar whatever the anticoagulant, a 1.5-fold higher abundance of chol-enriched domains was detected in citrate tubes. Therefore, values obtained on K^+^/EDTA tubes were multiplied by this factor. For chol-enriched domains, their abundance was either determined for the global hemi-RBC area or separately at HC and LC areas to distinguish domains mainly enriched with chol located in HC areas from those co-enriched with chol and polar lipids in LC areas (Conrard et al., 2018). The HC area corresponds to the periphery of spread RBCs while the LC area represents the centre of spread RBCs (Leonard et al., 2017b).

### EV and RBC membrane cholesterol content

Chol content was assessed using the Amplex Red cholesterol assay kit (Invitrogen) in the absence of chol esterase (Grimm et al., 2005; Tyteca et al., 2010). Washed RBCs were lysed in 1 mL of distilled water and diluted 8-fold in the kit reaction buffer while PBS-resuspended EVs were diluted 2-fold. Data on RBCs were normalised by the Hb content evaluated spectrophotometrically and expressed as % of fresh citrate blood tubes while data on EVs were expressed as a chol content (in µg) for 10^9^ EVs. Following an internal study in our laboratory, we noticed that the average RBC chol content was consistently higher in women than in men. As a result, the RBC chol content of the female reference control in the study was compared with the one of 2 female and 3 male donors. While the former was very close to the one measured for the two other female donors, it was 1.3-fold higher compared with average RBC chol content for the male donors (data not shown). Data obtained on lysed RBCs from male RCC donors were then corrected by this factor.

### RBC membrane microviscosity

RBC membrane microviscosity was studied by fluorescence lifetime imaging (FLIM) technique using a molecular rotor (BODIPY-C10), whose fluorescence lifetime is dependent on the microviscosity of the environment. RBCs were labelled in suspension for 60 min at 37°C with 1 µM of BODIPY-C10 dissolved in 1 mg/mL DMEM-BSA. After incubation, RBCs were washed in fresh DMEM, dropped off in plastic chambers (ibidi) for 8 min at RT. Cells were observed with the Zeiss LSM980 multiphoton microscope, which was equipped with a time-correlated single-photon counting (TCSPC) FLIM module (PicoQuant) for high-resolution microscopy. BODIPY-C10 was excited by a coherent (Chameleon Discovery) pulsed laser (80 MHz) at 800 nm. The emission was captured with a 505–545 nm bandpass filter at a resolution of 512 × 512 pixels. The fluorescence lifetimes for each pixel of the image corresponding to the cells were recorded to create the FLIM images. A minimum of 1,000 photons in the brightest pixel were acquired before stopping the FLIM acquisition. The FLIM images were analyzed using the SymPhoTime64 software (PicoQuant, Germany).

### Western blotting

After isolation, EV samples were resuspended in RIPA lysis buffer. RBC ghosts and human platelet lysates were prepared as in (Prausnitz et al., 1993; Octave et al., 2021) respectively. Equal protein amounts (except for plasma samples and platelet lysates) were diluted in a buffer containing 10 mM of dithiothreitol (DTT) and then loaded for sodium dodecylsulfate polyacrylamide gel electrophoresis (Mini-Protean TGX Precast Gels 4%–15% (w/v) SDS-PAGE; BioRad or Novex 4–12% Tris-Glycine Gels, Invitrogen). Then, proteins were transferred to polyvinylidene fluoride (PVDF) membranes and blocked for 2 h. Membranes were incubated overnight with anti-apolipoprotein B100 (Apo B100; BioConnect, SC-13538; 1:500), anti-apolipoprotein A1 (Apo A1; BioConnect, SC-376818; 1:500), anti-CD41 (Abcam, ab134131; 1:2,000), anti-glycophorin A (GPA; Merck, MABF758; 1:1,000), anti-flotillin 1 (BD Biosciences, BD610820; 1:500), anti-ankyrin (Merck, MAB1683; 1:1,000), anti-spectrin (α and β) (Merck, S3396; 1:500), anti-stomatin (Abcam, ab67880; 1:500) or anti-band3 (Invitrogen, MA1-20211; 1:4,000) antibodies. Secondary peroxidase-conjugated goat anti-rabbit or anti-mouse IgGs were then incubated for 1 h and washed. Signal revelation was performed with SuperSignalTM West Pico or Femto (ThermoScientific).

### Hemolysis and extracellular potassium and glucose measurements

RCCs were centrifuged at 2,000 g for 10–15 min and the supernatant was collected for Hb and extracellular K^+^ content for measurement with the ABL-90. As the device is calibrated for fresh blood samples, RCC supernatants were diluted in SAGM solution if necessary and data corrected for the dilution factor. The physiological range of plasma K^+^ levels corresponded to the values provided in the ABL-90 manual. For hematocrit, total Hb and glucose contents, RCCs were directly injected into the device. As RCCs contain the glucose-rich additive SAGM solution, the comparison with blood tubes was not relevant. Data were expressed in mmol/L for K^+^ and in mg/dL for glucose. The percentage of hemolysis was calculated as follows: (100-hematocrit) x supernatant Hb/total Hb.

### Data presentation and statistical analysis

**FIGURE 1:**
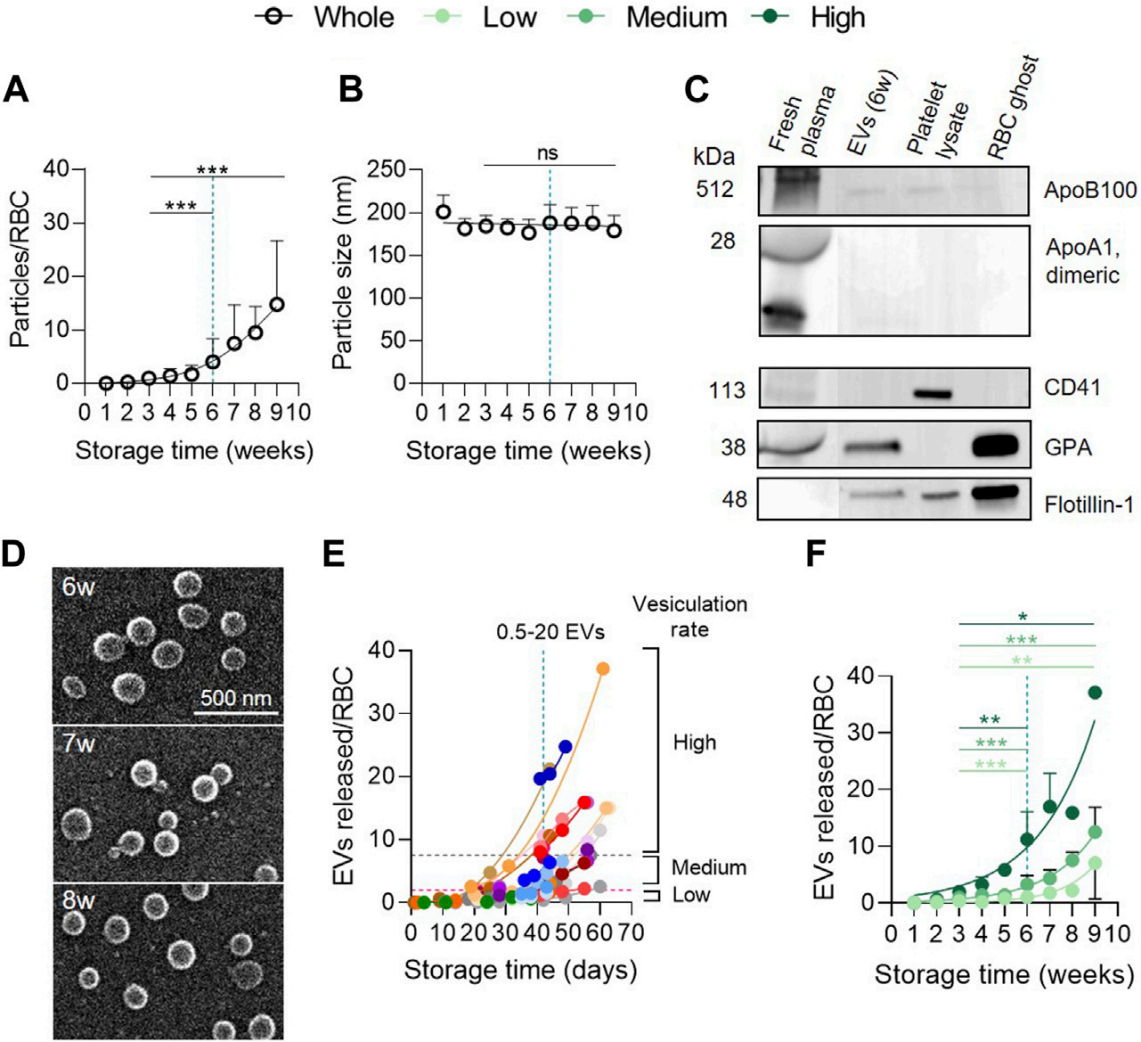
RBC vesiculation increases exponentially during storage whatever the donor cohort. **(A,B)** Particle abundance and size in the whole red blood cell concentrate (RCC) cohort. RCCs were centrifuged at low speed to separate cells from supernatants. Then, resulting supernatants were ultracentrifuged to pellet particles. Particle concentration and size were determined with NTA. The concentration was converted into a number of particles expressed in relation to the number of RBCs and the size was expressed in nm. The maximum legal storage period of 6 weeks (w) is indicated by a vertical blue dotted line. **(A)**, Number of particles expressed in relation to the number of RBCs at each week of storage from the 38 RCCs included in the study. Data are expressed as mean ± SD (open symbols). Unpaired *t*-test. Statistical significance is indicated above a line connecting 2 time intervals. **(B)**, Size of particles in nm (n = 38 RCCs). Data are expressed as mean ± SD. Unpaired *t*-test. **(C)** Purity of particle preparations. Western blotting for lipoprotein (apolipoprotein B100 (Apo B100) and apolipoprotein A1 (ApoA1)) and platelet (CD41) contaminations as well as for the presence of RBC (glycophorin A, GPA) and vesicle (flotillin-1) markers. 20 µg/well of particles and RBC ghost proteins. ApoB100 and ApoA1 were revealed on the same cut membrane. CD41 and GPA as well. Flotillin-1 was revealed after GPA membrane stripping. Fresh plasma from blood tubes, platelet lysates and RBC ghosts were used as positive controls. **(D)** Particle morphology. Particles isolated from 2 RCCs stored for the indicated times were laid down on poly-L-Lysine (PLL)-coated coverslips, prepared for and analysed by scanning electron microscopy. **(E)** Comparison of the 38 RCCs for kinetics of EV release during storage. One concentrate, one color. The vesiculation groups were arbitrarily established based on the EV level at 6w of storage: ‘high’ level with a vesiculation rate ≥7.5 EVs/RBC (area above the grey horizontal dotted line), ‘medium’ level with a vesiculation rate >2–7.4 EVs/RBC (area between the grey and pink horizontal dotted lines) and ‘low’ level with a vesiculation rate 0–2 EVs/RBC (area below the pink horizontal dotted line). **(F)** Classification of the whole RCC cohort into 3 groups based on the extent of vesiculation. High level (dark green, n = 9 RCCs), medium level (intermediate green, n = 20 RCCs), low level (light green, n = 9 RCCs). Data are expressed as mean ± SD. Mann Whitney test.

Kinetics during storage were represented in the form of weekly storage intervals (except in Figure 1E; Supplementary Figure S2; Supplementary Figure S8). The end of the legal storage period is indicated by a vertical blue dotted line. Horizontal black dotted line indicates reference values obtained from fresh blood tubes, mainly citrate-coated tubes except otherwise stated. The physiological range for metHb and K^+^ content in blood is represented by a grey frame while the maximal percentage of hemolysis authorized by the Council of Europe guidelines and the glucose concentration in SAGM and plasma are indicated by horizontal red full and dotted lines. Whenever data for a specific parameter was collected within one storage week from more than one RCC, the mean ± SEM (for most parameters) or ± SD (for EV abundance and size measurements, metHb content, hemolysis and extracellular K^+^ and glucose concentrations) for the concerned RCCs was calculated and represented on graphs. Notice that, in some conditions, the error bars are smaller than the size of the symbols.

In Figure 1E, data are presented for each RCC individually through color associations and in the form of the precise storage days in order to identify the ‘high’, ‘medium’ and ‘low’ vesiculation groups. The 3 groups were defined arbitrarily based on the number of EVs released at the legal storage period (42 days). The ‘high’ vesiculation group contains RCCs with a vesiculation rate ≥7.5 EVs/RBC at 42 days of storage (area above the horizontal grey dotted line), the ‘medium’ group includes RCCs with >2–7.4 EVs/RBC (area between horizontal pink and grey dotted lines) and the ‘low’ vesiculation group is composed of RCCs with 0–2 EVs/RBC (area below the horizontal pink dotted line). These groups consisted of 9, 20 and 9 RCCs respectively. To distinguish the different groups, the entire RCC cohort is represented by black opened circles, the high vesiculation by dark green closed circles, the medium vesiculation group by intermediate green closed circles and the low vesiculation group by light green closed circles. In Supplementary Figure S1, data from blood tubes were represented by squares.

To compare the 3 vesiculation cohorts at one specific time point, one-way ANOVA followed by Tukey’s multiple comparisons test or Kruskall-Wallis test with Dunn’s multiple comparison was performed. The statistical significance of these tests is presented in Supplementary Table S1. For the comparison of 2 time points during storage, an unpaired *t*-test (with Welch’s correction if required) or a Mann-Whitney test was performed. Graphically, statistical significance is indicated above a horizontal full line connecting 2 time intervals. Statistical comparison was mainly performed between 3w (corresponding to the storage interval in which RCCs are mostly transfused) and 6w (the maximal legal storage period) or between 3w and 9w (the longest storage period in this study). When necessary and when presenting similar behavior upon time, data from 2 and 3w or 9 and 10w were merged to get sufficient data for statistical analysis. Furthermore, if no data could be collected for these intervals, data from the neighboring intervals were used instead. Finally, to determine whether a difference exists between a reference value/internal control and a specific time point during storage or between a control and a treatment or to evaluate whether the reference blood tube donor is representative of 3 to 5 others, one sample *t*-test or Wilcoxon signed rank test was realized. Graphically, statistical significance is indicated in orange above one specific time interval to give comparison with the reference value/internal control (horizontal black dotted lines). Logarithmic transformation of particles/RBC, ROS, SM-enriched domains/hemi-RBC and the percentage of hemolysis was realized before the statistical test in order to fulfil normality. ***, *p* < 0.001; **, *p* < 0.01; *, *p* < 0.05; ns, not significant.

For correlations, data were transformed when necessary to ensure linearity. Correlation coefficients higher than 0.6 were plotted on graphs.

## Results

### Study design

A total of 38 RCCs were included into the study and followed for different parameters up to 10w. However, it should be noted that due to specific experiment timelines, all parameters were not assessed on the overall RCC cohort. In addition, 7 RCCs were freshly delivered from La Croix-Rouge de Belgique to our laboratory and evaluated from the first day of storage while 3 RCCs were delivered after 2w of storage, 12 RCCs after 3w, 5 RCCs after 4w, 4 RCCs after 5w and 7 RCCs after 6w. Since the overall RCC cohort could not be studied at all time points, data for most parameters were expressed in relation to values obtained on fresh blood tubes from the same control donor (represented on graphs by horizontal black dotted lines). In this manner, we avoided data misinterpretation due to experimental variability and all RCCs were compared with the same basal level. However, as few is known about control donor RBC membrane parameters routinely measured in our laboratory, we ensured that these parameters were not significantly different between the reference blood tube donor and 3 to 5 additional tube donors (Supplementary Figure S1).

### RBC concentrates can be divided into 3 groups based on their extent of vesiculation

Particles in RCC supernatants were concentrated by differential ultracentrifugation and measured by NTA for their abundance and size. As previously shown (Rubin et al., 2008; Roussel et al., 2017; Lauren et al., 2018), the number of particles, expressed in relation to the number of RBCs present in RCCs, increased exponentially during storage, revealing significant differences between 3 and 6w and between 3 and 9w (Figure 1A). However, the size remained stable over storage, at around 180 nm (Figure 1B). At 6w, a mean of 4 particles for one RBC was measured (Figure 1A), corresponding to ∼7 × 10^12^ particles in a 250 mL bag. In reality, this number showed a ∼40-fold variability (∼0.5–20 particles/RBC) between RCCs. This difference did not result from measurement irreproducibility by NTA, since 4 RCCs which originated from the same 2 donors (2 RCCs from a single donor) showed a similar particle abundance during storage (Supplementary Figure S2). A potential contamination of samples was also excluded, as shown by very few amounts of lipoproteins and platelets (or their vesicles) compared with fresh plasma (Figure 1C; Supplementary Figures S3A, B). Inversely, particles appeared to have an erythrocytic origin, as revealed by the detection of the RBC marker GPA, and exhibited the raft marker flotillin-1, known to be associated with EVs (Salzer and Prohaska, 2001; Freitas Leal et al., 2020). Further analysis with electron microscopy showed vesicle-like structures with a spherical morphology and no aggregates (Figure 1D). Altogether these data indicated that particles were mainly, if not exclusively, EVs and that RCCs released a variable quantity of vesicles upon storage which cannot be explained by technical issues.

To further determine the origin of this variability, RCCs were separated into 3 groups based on their vesiculation rate at the legal storage period of 6w: ‘low’ rate (0–2 EVs/RBC), ‘medium’ rate (>2–7.4 EVs/RBC) or ‘high’ rate (≥7.5 EVs/RBC) (Figures 1E, F). These groups consisted of 9, 20 and 9 RCCs respectively. Each cohort showed an exponential and significant increase of EVs between 3 and 9w (Figure 1F) but was significantly different from each other in terms of amounts of EVs released (Supplementary Table S1, first column). This variability could not be attributed to basic quality measures such as hemolysis and extracellular K^+^ and glucose contents, since these parameters were in the expected range and were not different from one cohort to another (Supplementary Figure S4).

### Spherocytes appear late during storage and correlate positively with the extent of EV release

**FIGURE 2:**
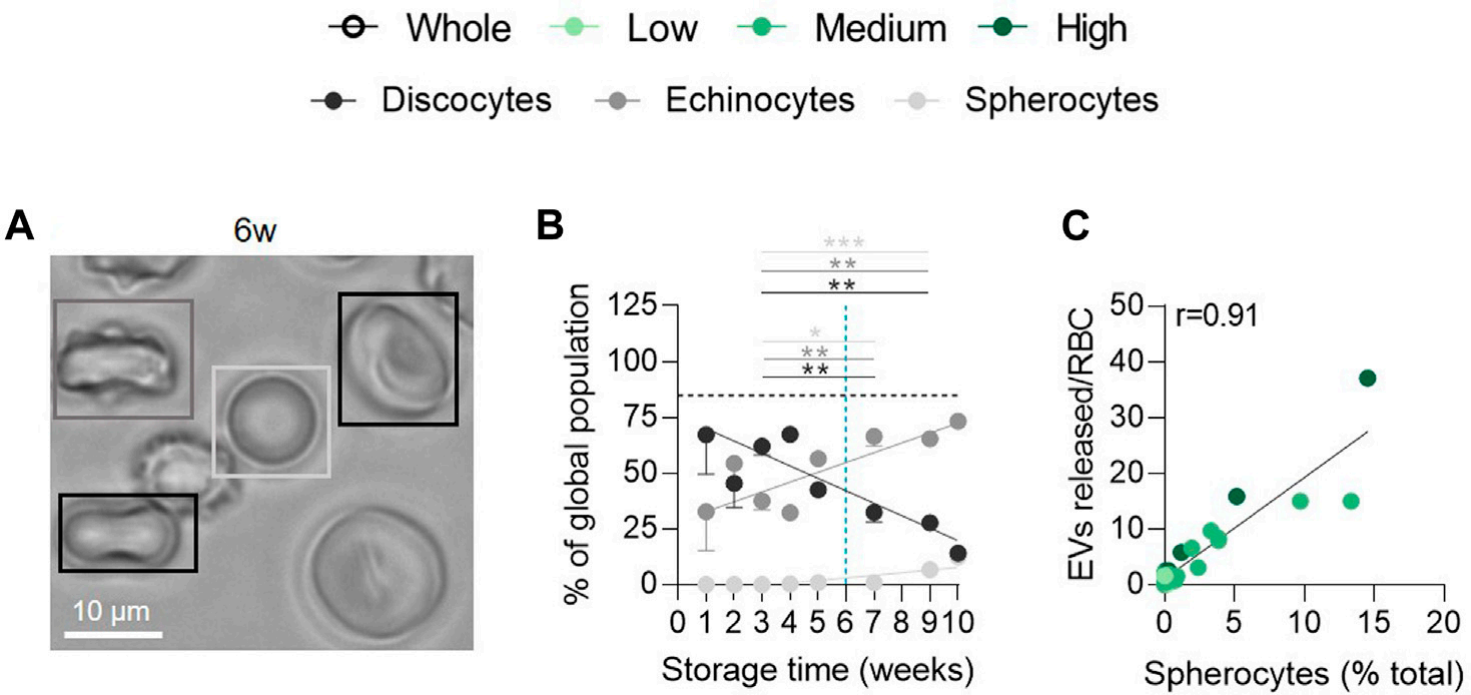
The late appearance of spherocytes during storage correlates positively with the extent of EV release. RBCs were fixed in suspension, placed on PLL-coated coverslips and observed by light microscopy. **(A)** Representative image of RBC morphology at 6w of storage. **(B)** Quantification of the relative abundance of discocytes (black), echinocytes (dark grey) and spherocytes (light grey) and expression as % of the global RBC population (n = 15 RCCs). The horizontal black dotted line represents the discocyte abundance of the fresh blood tube. Data are expressed as mean ± SEM. Unpaired *t*-test. **(C)** Correlation between the number of EVs released per RBC and the % of spherocytes in the global population.

Since vesiculation is usually associated with RBC morphological modifications (D’alessandro et al., 2012; Roussel et al., 2017; Alaarg et al., 2013), we next evaluated the relative proportion of discocytes, echinocytes and spherocytes upon time. The abundance of discocytes was already less important than in fresh blood tubes at 1w and further decreased upon storage in favour of echinocytes and a minority of spherocytes (Figures 2A, B). The decrease of discocytes and the increase of spherocytes both correlated with the rise of released EVs for all vesiculation cohorts (data not shown and Figure 2C), suggesting that the relatively late appearance of spherocytes from 6w of storage could be attributed to vesiculation (Roussel et al., 2017).

### Intracellular ATP concentration is maintained higher than in fresh tubes until 4 weeks and do not differ at 6 weeks between the 3 vesiculation cohorts

**FIGURE 3:**
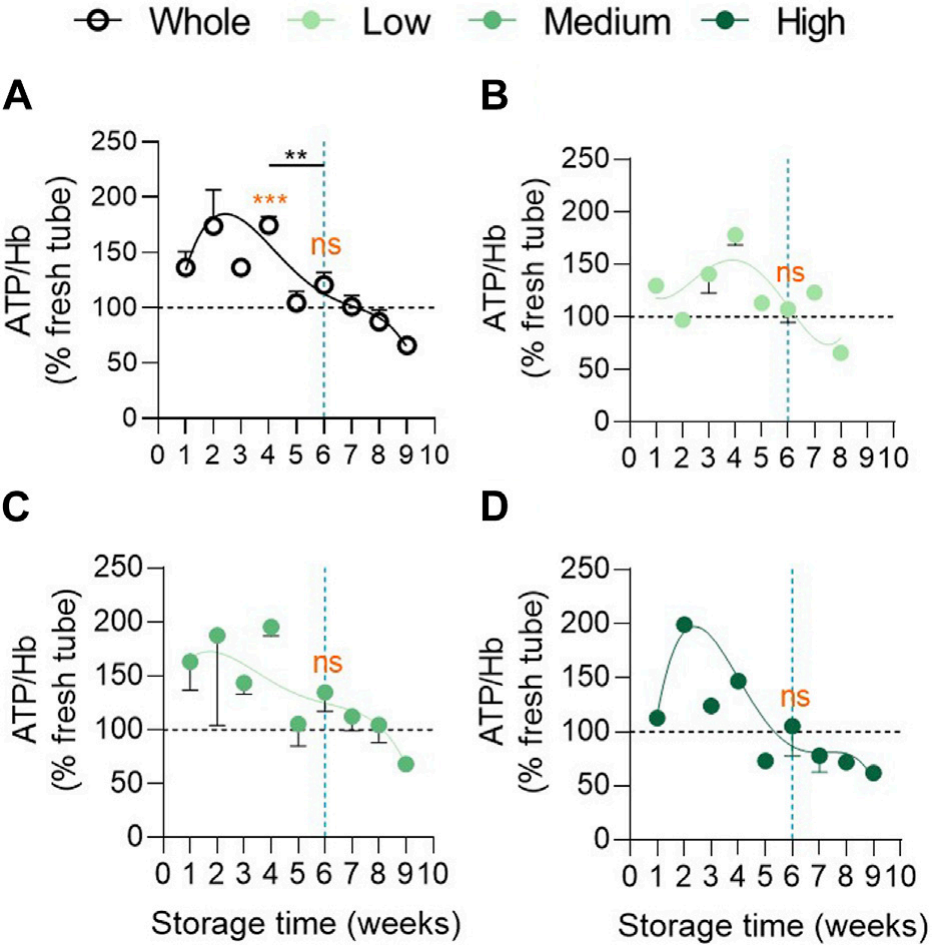
Intracellular ATP concentration remains higher in the 3 vesiculation groups in comparison with fresh tubes until 4 weeks of storage and does not differ at 6 weeks. Intracellular ATP concentration was assessed with the luminescent ATP detection assay kit, normalized on the haemoglobin (Hb) content and expressed in % of fresh RBCs in blood tubes. Reference values from fresh blood tubes are represented by horizontal black dotted lines. Evolution of the ATP concentration during storage in the overall RCC population (**(A)**, n = 24 RCCs) and in the 3 cohorts of vesiculation (**(B)**, n = 7; **(C)**, n = 11; **(D)**, n = 6 RCCs). Data are expressed as mean ± SEM. Statistical significance of unpaired *t*-test is indicated above a line connecting 2 time intervals while one sample *t*-test (for the entire cohort) or Wilcoxon signed rank test (for each cohort individually) are indicated in orange above one time interval to give comparison with the internal control (fresh blood tubes).

We next wondered whether the high variability in EV abundance between groups could be explained by a differential intracellular ATP concentration. Indeed, ATP levels are known to decrease during storage leading to reorganisation of cytoskeleton, reduction of the antioxidant system activities and EV formation (Yoshida et al., 2019). In the overall RCC population, intracellular ATP levels first increased, showing a peak between 1 and 4w of storage, and then decreased progressively (Figure 3A), as observed by Gevi et al. (Gevi et al., 2012). Despite differential kinetics, no significant difference in ATP level could be detected at 6w between groups (Figures 3B–D; Supplementary Table S1, second column). Compared with fresh tubes (horizontal black dotted line), ATP levels in RCCs stayed higher until 4w whatever the cohort (Figure 3). This could be due to the fact that, despite a constant decrease, the glucose content in SAGM never dropped to the plasma level during the whole storage period in each cohort (Supplementary Figures S4I–L). Taken together, these data indicated that the EV abundance variability between the 3 cohorts at 6w could not be explained by a differential intracellular ATP content.

### Reactive oxygen species and methaemoglobin do not accumulate during storage in the 3 vesiculation cohorts

**FIGURE 4:**
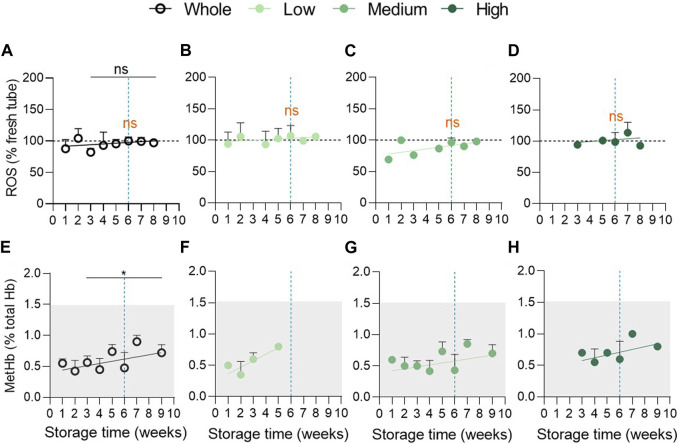
Oxidative stress in the form of reactive oxygen species and methaemoglobin does not accumulate during storage. **(A–D)** Intracellular reactive oxygen species levels were detected with the 2,7-dichlorodihydrofluoresceindiacetate (H_2_DCFDA) probe in RCCs and in fresh blood tubes and analysed by flow cytometry. The median fluorescence intensity (MFI) for RCCs was expressed as % of the MFI calculated for blood tubes (horizontal black dotted lines). Evolution upon time of the intracellular ROS content in the overall population (**(A)**, n = 17 RCCs) and in the 3 vesiculation cohorts (**(B)**, n = 5; **(C)**, n = 8; **(D)**, n = 4 RCCs). Data are expressed as mean ± SEM. Statistical significance of unpaired *t*-test is indicated above a line connecting 2 time intervals while one sample *t*-test (for the entire cohort) or Wilcoxon signed rank test (for each cohort individually) are indicated in orange above one time interval to give comparison with the internal control (fresh blood tubes). **(E–H)** Intracellular metHb concentration was assessed with the automated blood gas analyser ABL-90 in RCCs and in fresh blood tubes. The metHb amount was expressed as % of total Hb levels. The physiological range of metHb levels is indicated by the clear grey frame. Evolution upon time of the intracellular metHb content in the overall population (**(E)**, n = 13 RCCs) and in the 3 vesiculation cohorts (**(F)**, n = 3; **(G)**, n = 8; **(H)**, n = 2 RCCs). Data are expressed as mean ± SD. Mann-Whitney test (for the entire cohort).

As oxidative stress is proposed to be a key player in the development of storage lesions (D'Alessandro et al., 2012; Kriebardis et al., 2008; Gevi et al., 2012; Delobel et al., 2012; Yoshida et al., 2019), we next measured intracellular ROS and metHb contents. Surprisingly, the ROS content was similar in fresh concentrates and in blood tubes and was not modified upon storage (Figure 4A). MetHb levels showed a tendency of constant increase upon storage but stayed in the normal range for fresh blood tubes as defined by the manufacturer of the automated blood gas analyser ABL-90 (grey area at Figure 4E). A technical issue was excluded as ROS and metHb highly increased under H_2_O_2_ treatment (Supplementary Figure S5) and during the storage of K^+^/EDTA blood tubes (Cloos et al., 2020). Moreover, even after cohort division, we were not able to detect accumulation of ROS (Figures 4B–D) or metHb (Figures 4F–H) in RCCs. Overall, oxidative stress did not appear to develop in RCCs at least in the form of ROS and metHb and can therefore not reflect the differences in vesiculation between the 3 groups.

### Spectrin occupancy is impaired early during storage and differentially in the 3 vesiculation cohorts

**FIGURE 5:**
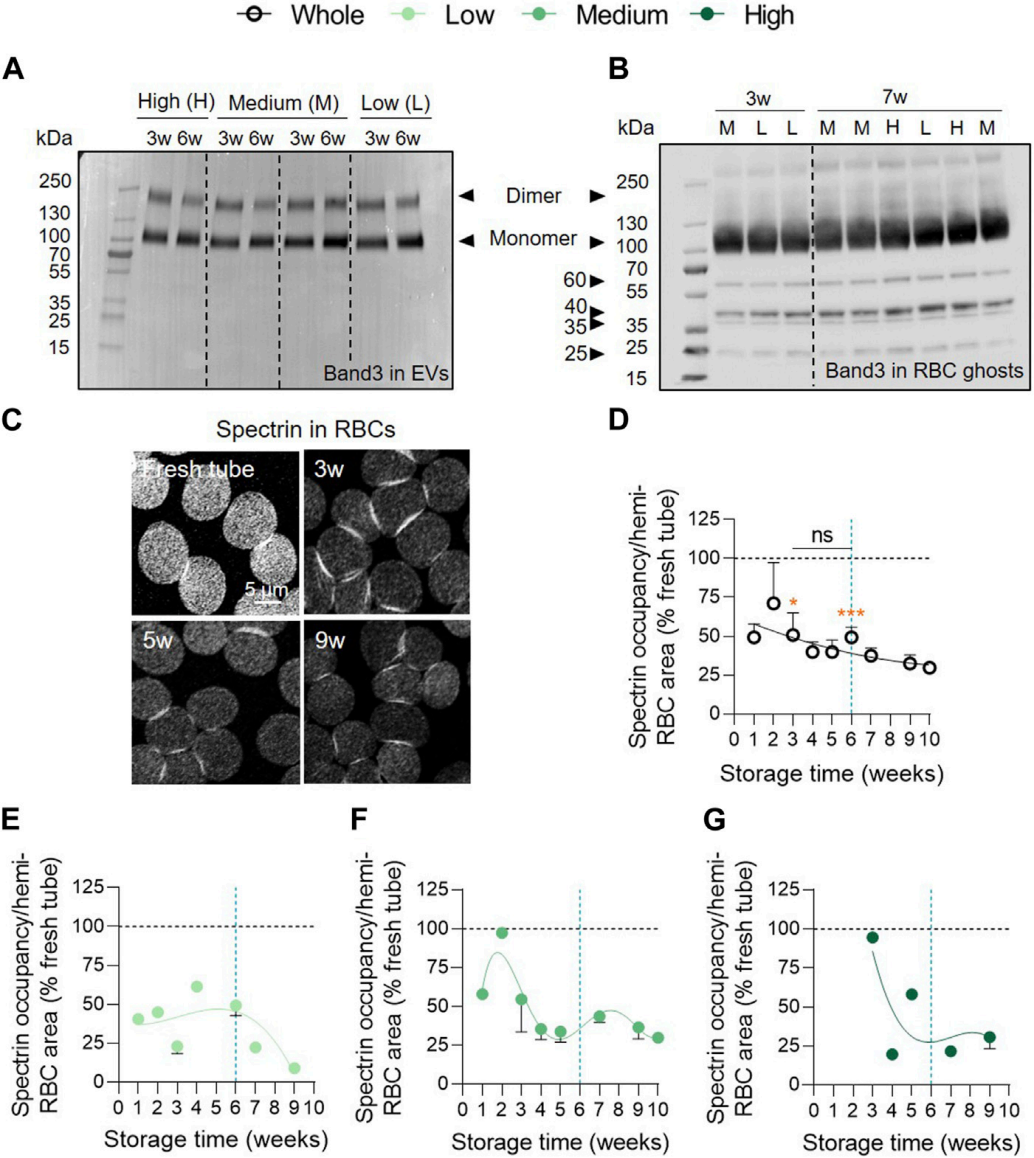
The cytoskeleton membrane occupancy is altered early but differentially in the 3 vesiculation groups in contrast to band3 integrity. **(A)** Band3 in EVs. Representative Western blot for the presence of band3 in EVs at 3 and 6w in the 3 vesiculation cohorts (n = 4 RCCs; 1, 2 and 1 RCC(s) from the low, medium and high vesiculation cohort, respectively). 2.4 µg/well of EV proteins. **(B)** Band3 in RBC ghosts. Representative Western blot for the presence of band3 in RBC ghosts at 3 and 7w in the 3 vesiculation cohorts (n = 9 RCCs). 20 µg/well of RBC ghost proteins. L for RBC ghosts from the low vesiculation cohort, M for the medium and H for the high vesiculation cohort. **(C–G)** RBC spectrin membrane occupancy. RBCs were dropped off on PLL-coated coverslips, permeabilised, fixed, immunolabelled for α,β-spectrin and analysed by confocal microscopy. Spectrin-membrane occupancy was determined with Fiji software and expressed as % of fresh blood tubes (horizontal black dotted lines). **(C)**, Representative images of spectrin membrane occupancy during storage. Kinetics of spectrin membrane occupancy during storage in the overall RCC population (**(D)**, n = 17 RCCs) and in the 3 cohorts of vesiculation (**(E)**, n = 4; **(F)**, n = 10; **(G)**, n = 3 RCCs). Data are expressed as mean ± SEM. Statistical significance of unpaired *t*-test is indicated above a line connecting 2 time intervals while one sample *t*-test is indicated in orange above one time interval to give comparison with the internal control (fresh blood tube).

Intrigued by the absence of ROS accumulation, we next wondered whether these reactive species could have attacked other targets than Hb, i.e., the anchorage complex-associated anion transporter band3 and the cytoskeletal protein spectrin (Antonelou et al., 2010; Rinalducci et al., 2012; Prudent et al., 2018), thereby preventing their accumulation and detection. We started by evaluating the presence of band3 in EVs at 3 and 6w of storage by Western blotting in reducing conditions for the 3 vesiculation cohorts. Band3 was found in EVs mainly in the form of monomers but also in dimers at both 3 and 6w whereas no degradation products were detected. However, no obvious differences were visible between 3 and 6w of storage or between the 3 vesiculation cohorts (Figure 5A). We then determined whether band3 dimers were also present in RBC ghosts for each vesiculation cohort. We observed that dimers detected in EVs are present in considerably lower proportions in RBC ghosts when compared with the monomeric form. However, degradation products at approximately 60, 40, 35 and 25 kDa could be visualised (Figure 5B). These band3 fragments were previously reported by Rinalducci et al. and were attributed to oxidative lesions (Rinalducci et al., 2012). Once again, no obvious changes in the profile of band3 could be noticed in the RBC ghosts regarding storage time or according to the vesiculation cohort. The spectrin network was then immunolabelled, visualised by confocal microscopy and quantified for membrane surface occupancy. In the overall RCC population, spectrin occupancy was significantly 2-fold lower than in blood tubes and did not considerably evolve over time (Figures 5C, D). Once cohorts were separated, spectrin occupancy tended to decrease between 2–3 and 5w in the medium and high vesiculation cohorts. In contrast, the low vesiculation cohort showed a stable spectrin occupancy until 6w and then started to decrease (Figures 5E–G). Altogether, these data suggested that the membrane:cytoskeleton interactions were impaired early during storage which could in part be attributed to oxidative stress. However, whereas spectrin occupancy was differentially altered in the 3 vesiculation groups and evolved over time, it was not the case for band3 alterations.

### Membrane transversal asymmetry is altered late during storage and differentially in the 3 vesiculation cohorts

**FIGURE 6:**
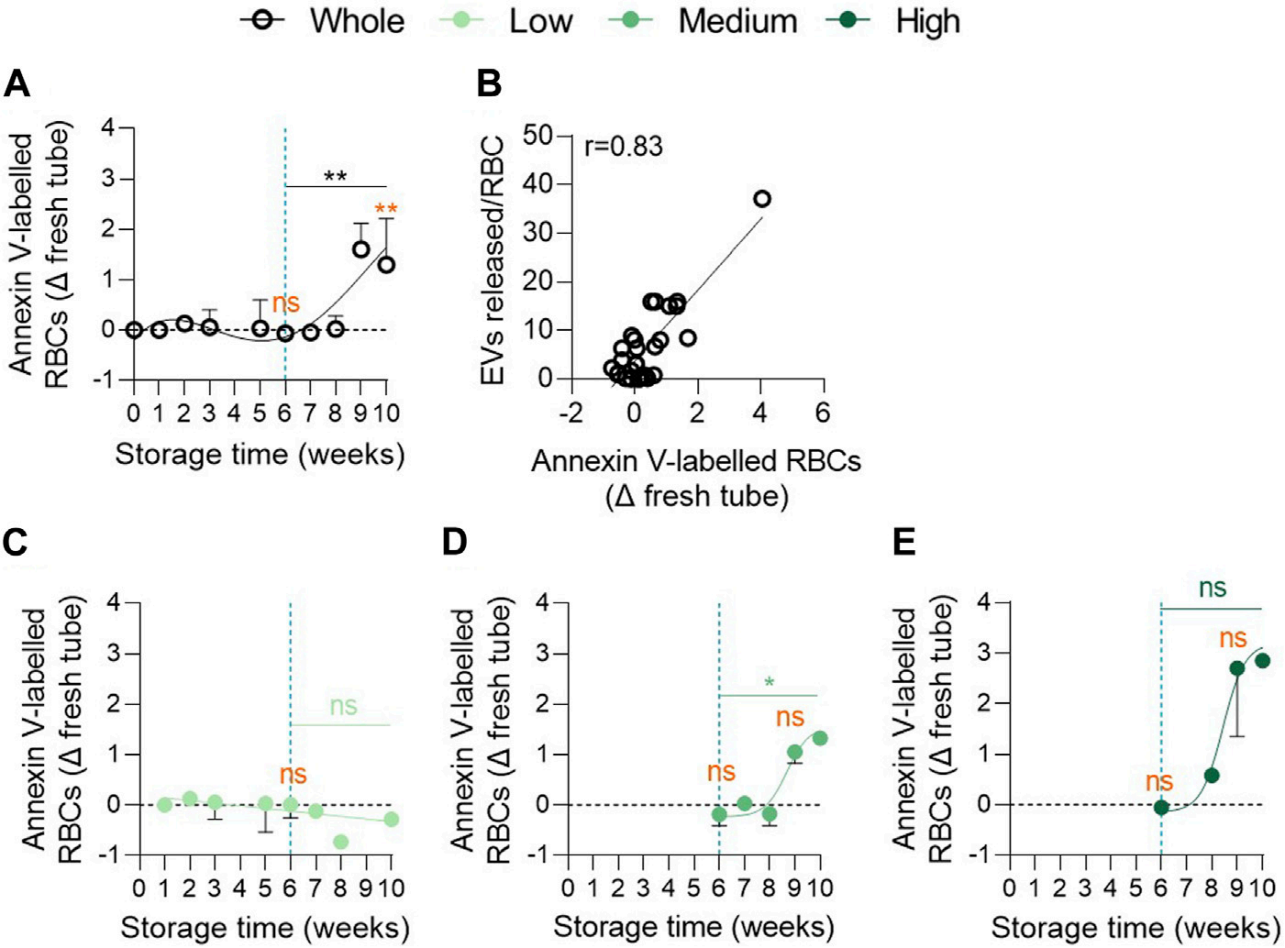
The proportion of phosphatidylserine exposing RBCs increases exponentially in the medium and high vesiculation groups from 8 weeks of storage. RBCs were labelled with Annexin-V-FITC and then analysed in flow cytometry. The % of Annexin V-FITC positive RBCs was determined by positioning the cursor at the edge of the labelled cell population for fresh blood tubes. The proportion of labelled RBCs in tubes was subtracted from the percentage of labelled cells in RCCs. **(A,C–E)** Number of PS-exposing RBCs upon time in the overall RCC population (**(A)**, n = 16 RCCs) and in each vesiculation cohort (**(C)**, n = 4; **(D)**, n = 8; **(E)**, n = 4 RCCs). Data are expressed as mean ± SEM. Statistical significance of unpaired *t*-test (for the entire cohort) or Mann-Whitney test (for each cohort individually) is indicated above a line connecting 2 time intervals. In orange, one sample *t*-test (for the entire cohort) or Wilcoxon signed rank test (for each cohort individually) are indicated above one time interval to give comparison with the internal control (fresh blood tube). **(B)** Correlation between EVs released per RBC and the % of Annexin-V-labelled RBCs.

To explore whether cytoskeleton alterations were associated with impairments of membrane transversal asymmetry (Manno et al., 2002), PS externalisation was determined by flow cytometry using Annexin-V labelling. Although PS exposure remained similar to that of fresh blood tubes until 6w of storage, a significant increase was noticed between 6 and 9w with ∼2% of PS-exposing RBCs at 9w (Figure 6A). This parameter correlated positively with EV release by RBCs (Figure 6B). More importantly, the 3 cohorts could be discriminated with this parameter. Indeed, the low vesiculation group did not expose PS whatever the storage duration while the medium and high vesiculation groups showed an increase from 8w of storage reaching ∼1.5% and ∼3% PS exposure at 10w, respectively (Figures 6C–E). Moreover, significant differences were obtained between the low and high groups at 9w of storage (Supplementary Table S1, third column). Taken together, our data revealed that the 3 cohorts could be distinguished by the extent of PS surface exposure. Nevertheless, the kinetics of changes were quite late, occurring during 7–10w. As PS-cytoskeleton interactions are known to modulate membrane stability (Manno et al., 2002), we next wondered whether lateral membrane heterogeneity in domains at the RBC surface could also be altered and contribute to the variability in EV release between groups.

### Sphingomyelin-enriched domains rise exponentially upon storage and differentially in the 3 vesiculation cohorts

**FIGURE 7:**
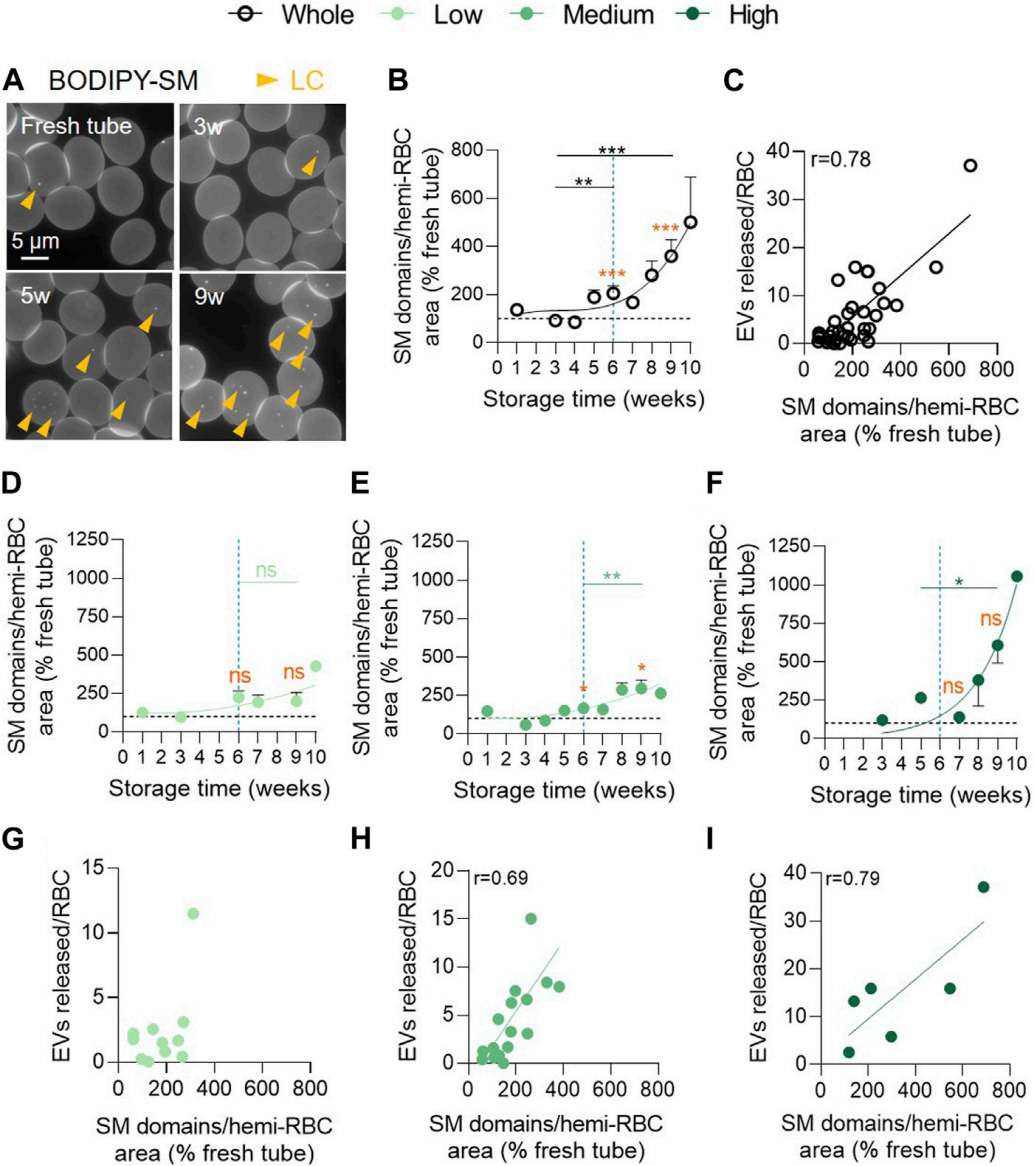
Sphingomyelin-enriched domain abundance increases exponentially in the medium and high vesiculation groups and positively correlates with EV accumulation. RBCs were immobilized on PLL-coated coverslips, labelled with BODIPY-SM and then observed by fluorescence microscopy. The domain abundance at the RBC surface in RCCs corresponds to the average number of domains per hemi-RBC area and expressed as % of domain abundance in fresh blood tubes. **(A)** Representative images upon time. Yellow arrowheads, lipid domains in low curvature (LC). **(B,D–F)** Evolution upon time of the number of SM-enriched domains from the overall RCC population (**(B)** n = 15 RCCs) and the 3 cohorts of vesiculation (**(D)**, n = 4; **(E)**, n = 8; **(F)**, n = 3 RCCs). Data are expressed as mean ± SEM. Unpaired *t*-test (for the entire cohort) or Mann-Whitney test (for each cohort individually). Statistical significance is indicated above a line connecting 2 time intervals. In orange, one sample *t*-test (for the entire cohort) or Wilcoxon signed rank test (for each cohort individually) are represented above a precise time interval to give comparison with the internal control (fresh blood tube). **(C,G–I)** Correlation between the number of EVs released by RBC and the SM-enriched domain abundance in the total RCC population **(C)** or in the 3 vesiculation groups **(G–I)**. Correlation coefficients are indicated only if > 0.6.

We therefore analysed the abundance of SM-enriched domains, mostly if not exclusively associated with the LC areas of RBCs (Leonard et al., 2017b). Until 4w of storage in the overall RCC population, this abundance remained stable and was relatively similar to fresh blood tubes. From 5w, SM-enriched domains started to significantly and exponentially increase (Figures 7A, B) and correlated positively with the amount of EVs released (Figure 7C). As for PS exposure, the low vesiculation cohort was protected from this membrane change and showed no correlation between the number of SM-enriched domains and EVs (Figures 7D,G). Conversely, the medium and high vesiculation groups showed a significant exponential increase of SM-enriched domains which correlated with the amount of EVs released (Figures 7E, F, H, I). Nevertheless, no significant difference between the 3 groups could be detected at 9w (Supplementary Table S1, fourth column).

### The decrease of cholesterol-enriched domains from the RBC surface upon storage occurs with differential kinetics in the 3 vesiculation cohorts and is accompanied by an increase of cholesterol in EVs

**FIGURE 8:**
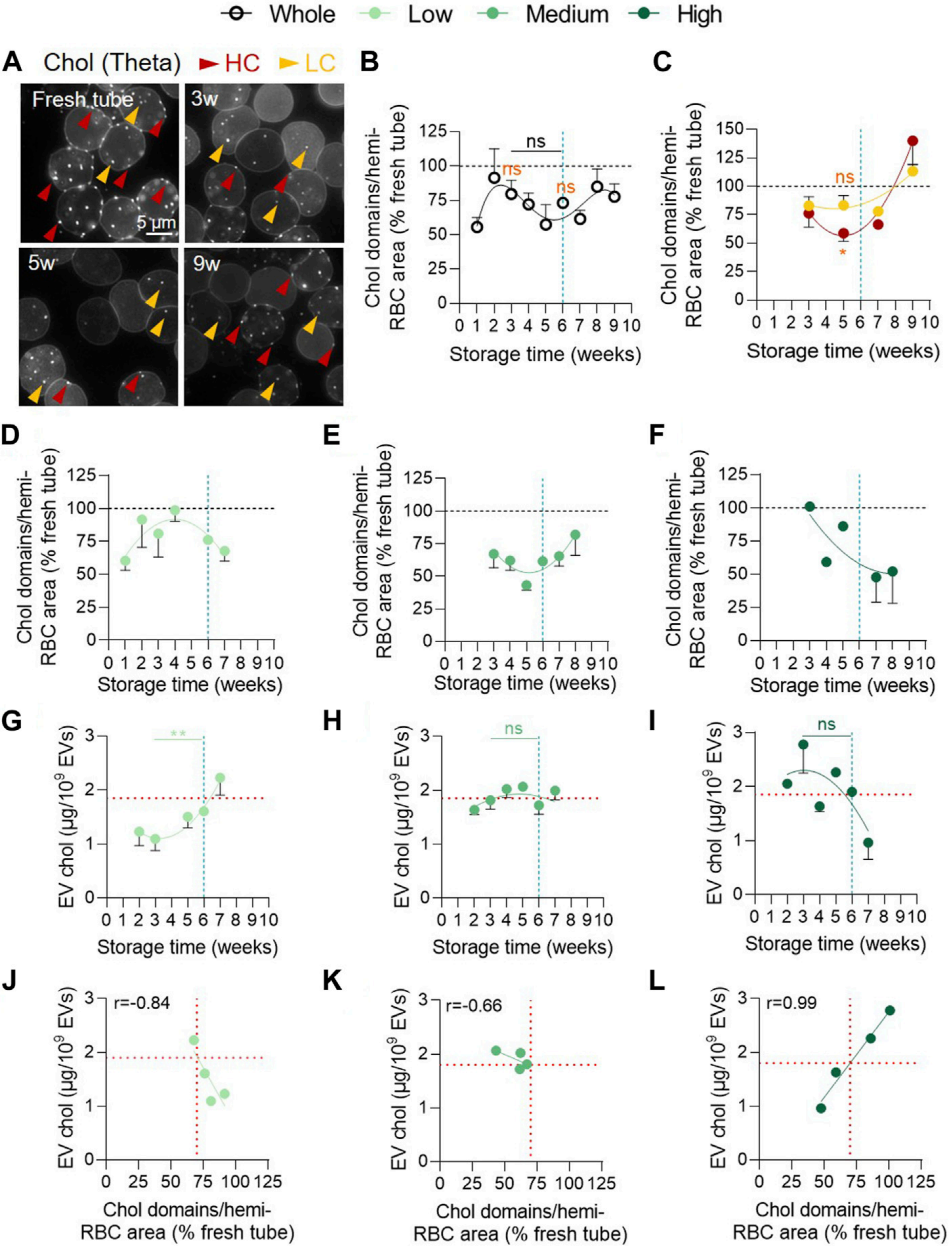
The decrease of cholesterol-enriched domain abundance from the RBC surface and the cholesterol association with EVs are observed in the 3 vesiculation cohorts but with differential kinetics. **(A–F)** Abundance of cholesterol (chol)-enriched domains at the RBC surface. RBCs were labelled in suspension with the fluorescent Theta toxin fragment, immobilized on PLL-coated coverslips and then observed by fluorescence microscopy. The domain abundance at the RBC surface in RCCs corresponds to the average number of domains per hemi-RBC area and expressed as % of domain abundance in fresh blood tubes. **(A)** Representative images upon time. Red arrowheads, lipid domains in high curvature (HC) area; yellow arrowheads, lipid domains in low curvature (LC) area. **(B,C)** Kinetics upon storage of the abundance of total chol-enriched domains (**(B)**, n = 18 RCCs) or chol-enriched domains associated with either RBC HC areas (red) or LC areas (yellow) from the overall RCC cohort (**C**, n = 6 RCCs). Data are expressed as mean ± SEM. Mann-Whitney test. Statistical test is indicated above a line connecting 2 time intervals. In orange, Wilcoxon signed rank test is represented above a precise time interval to give comparison with the internal control (fresh blood tube). **(D–F)**, Kinetics of the abundance of content chol-enriched domains in the 3 vesiculation groups (**(D)**, n = 6; **(E)**, n = 8; **(F)**, n = 3 RCCs). Data are expressed as mean ± SEM. **(G–I)**. Evolution of chol content in EVs upon time in the 3 groups of vesiculation (**(G)**, n = 7; **(H)**, n = 16; **(I)**, n = 8 RCCs). Chol was evaluated using the Amplex Red cholesterol assay kit. Data were expressed as a quantity of chol (in µg) for 10^9^ EVs and presented as mean ± SEM. Mann-Whitney test. **(J–L)** Correlation between the EV chol content and the abundance of chol-enriched domains in each vesiculation cohort. Horizontal red lines in G-L indicate the EV chol content upon a ~30% decrease of chol-enriched domain abundance.

Next, we evaluated the abundance of chol-enriched domains at the RBC surface in RCCs by fluorescence microscopy. We observed a complex non-significant kinetics with a first transient increase around 2w of storage followed by a decrease between 2 and 5w and a re-increase from 6w of storage. Of note, the number of chol-enriched domains remained lower than in fresh RBCs from blood tubes for the whole storage period (Figures 8A,B). As no statistical difference during storage could be evidenced, we tried to determine from which membrane region the decrease of chol-enriched domains between 2 and 5w could occur. Indeed, we previously showed that lipid domains are preferentially lost from HC areas in K^+^/EDTA tubes upon storage (Leonard et al 2017b; Cloos et al., 2020). We therefore differentiated and quantified the domain abundance in HC (red arrowheads) and LC areas (yellow arrowheads) for several RCCs. This analysis showed a significantly lower number of chol-enriched domains in HC but not in LC at 5w compared with fresh tube (Figure 8C), supporting our previous data in K^+^/EDTA tubes upon storage (Cloos et al., 2020) and suggesting that chol-enriched domains could be lost by vesiculation from HC areas.

An alternative explanation behind the absence of statistical differences during storage is that the 3 vesiculation cohorts could exhibit differential kinetics of chol-enriched domain alteration. We therefore analysed separately the evolution of chol-enriched domains in the 3 cohorts. Despite different behaviours, the 3 vesiculation groups exhibited a decrease in domain abundance at some time points (Figures 8D–F). To address whether this decrease could be associated with an increase of chol content in EVs, we first evaluated by Western blotting the EV content in stomatin, a chol-binding protein (Salzer et al., 2007). We showed that stomatin was the only protein enriched in EVs compared with RBC ghosts regardless of the storage time. GPA and spectrin were also present in EVs but not enriched compared with RBC ghosts and ankyrin could not be detected (Supplementary Figures S6A, B). Additionally, stomatin was more enriched in EVs than the raft marker flotillin-1 at 6w except for one RCC from the high vesiculation group (Supplementary Figures S6C, D). Second, we measured the chol content in EVs during storage in the 3 vesiculation groups. A mirror image between the evolution of chol-enriched domains and EV chol content was evident for the 3 groups (Figures 8D–I) and was reflected in the correlations between these two parameters (Figures 8J–L) suggesting that chol-enriched domains could contribute to vesiculation. However, while a negative correlation was observed for the low and medium vesiculation groups as expected, a positive correlation was seen in the high vesiculation group (Figures 8J–L), indicating that lower the abundance of chol-enriched domains, lower the chol content in EVs. Combined with the observations that this high vesiculation cohort presented the highest EV number released, the strongest chol level in EVs at 3w (Figures 8G-I) and a loss of chol content from the RBC membrane (Supplementary Figure S7), this positive correlation suggested that other membrane regions than chol-enriched domains are lost from RBCs in this group.

To confirm the potential contribution of the loss of chol-enriched domains to the vesiculation process whatever the cohort, we looked at the chol content in EVs of the 3 groups for a same chol-enriched domain loss of ∼30% (corresponding to the average drop observed for the entire cohort; see Figure 8B and vertical red dotted lines in Figures 8J–L). Such domain decrease was associated with a similar chol content of ∼1.9 µg per 10^9^ EVs in all 3 groups and was reached only after 6w of storage for the low vesiculation group, between 3 and 4w for the medium vesiculation group and as early as 2w for the high vesiculation group (horizontal red dotted lines in Figures 8G–I). Altogether, these data indicated that the decrease of chol-enriched domains was accompanied by an increase of chol in EVs, suggesting that chol-enriched domains were lost by vesiculation. Moreover, this decrease occurred at differential time points during the storage in the 3 vesiculation cohorts, providing an additional explanation for the non-significant kinetics of chol-enriched domains in the overall cohort besides the differential domain decrease from HC and LC areas.

### The RBC membrane microviscosity increases transiently during storage and precedes vesiculation

**FIGURE 9:**
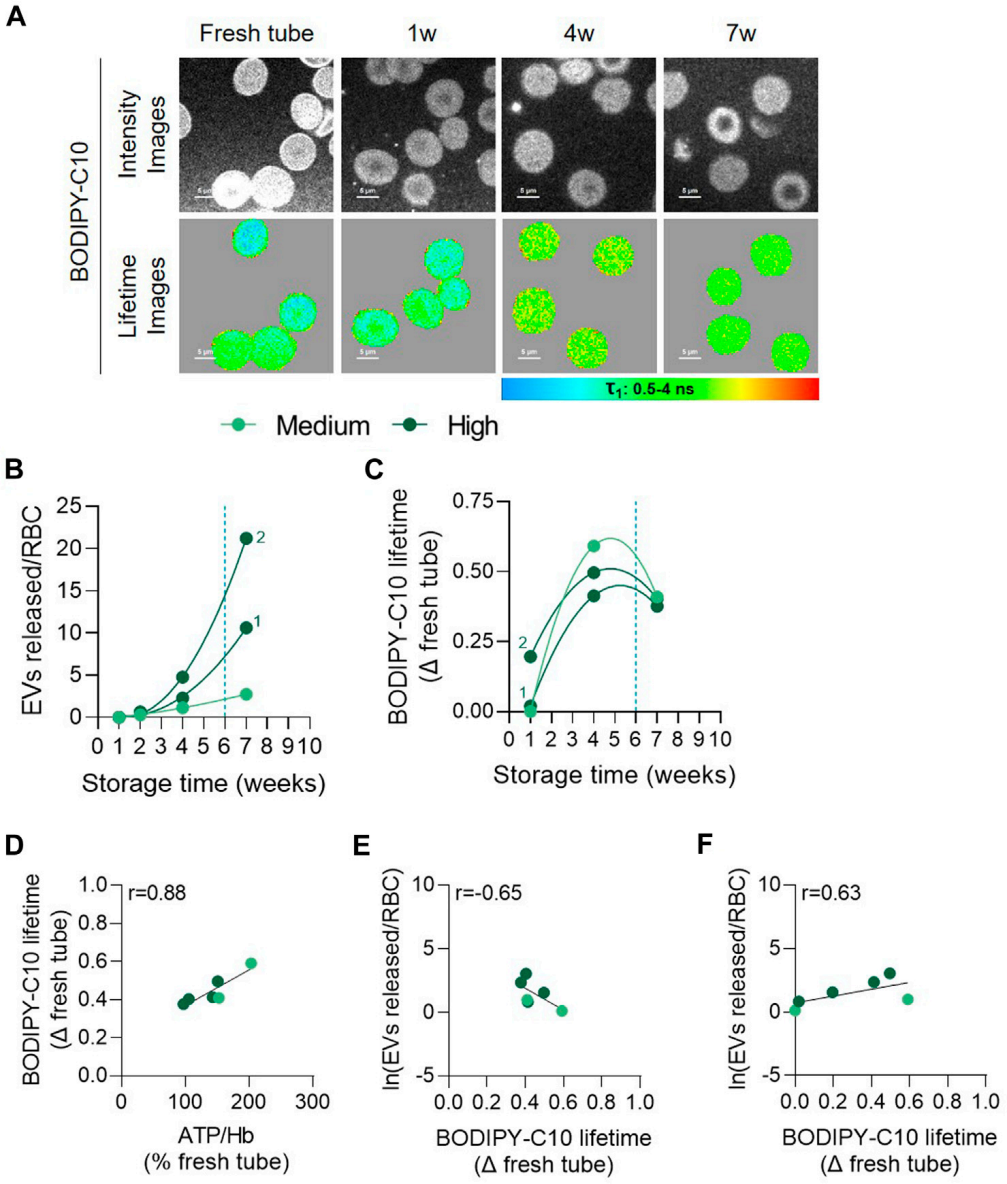
The membrane microviscosity increases early during storage and positively correlates with ATP and precedes EV release. RBCs from 3 RCCs were labelled in suspension with BODIPY-C10, washed, dropped off in plastic chamber and examined by confocal microscopy using multiphoton mode. Lifetimes of BODIPY-C10 were determined with SymPhoTime 64 software and expressed as a delta of fresh tube. **(A)** Representative images during storage. The lifetime values (τ) ranged from 0.5 to 4 ns and were shown in pseudocolor-coded images. The longer the lifetime, the higher the membrane microviscosity. **(B)** Number of EVs per RBC at each week of storage from 3 RCCs, 1 from the medium group and 2 from the high vesiculation group (distinguished by a number). **(C)** Evolution of BODIPY-C10 lifetime upon time in the 3 RCCs as a delta of fresh tube. **(D,E)** Correlation between the intracellular ATP content **(D)** and Napierian logarithm of EVs **(E)** with the BODIPY-C10 at the same time intervals. **(F)** Correlation between the Napierian logarithm of EVs and the BODIPY-C10 lifetime in RCCs realized by associating microviscosity at 1w with the EV level at 4w and the microviscosity at 4w with the EV level at 7w.

To then gain insight on the mechanism behind the decrease of chol-enriched domains in the 3 vesiculation groups while addressing the possibility of additional RBC membrane alterations in the high vesiculation group, we measured the RBC membrane microviscosity. For this purpose, fluorescence lifetime of BODIPY-C10 was monitored at 1, 4 and 7w of storage of RBCs from 1 medium and 2 high vesiculation RCCs (Figures 9A, B). The lifetime of this probe correlates with the viscosity of its environment (Mozaheb et al., 2022), a longer lifetime indicating a higher microviscosity of the membrane. After labelling, RCCs were observed by multiphoton microscopy and analysed for the BODIPY-C10 lifetime. Data were expressed as a difference of BODIPY-C10 lifetime *versus* fresh blood tubes. The increase in membrane microviscosity as compared with RBCs from fresh tubes was already present after 1w in the RCC exhibiting the highest vesiculation rate (characterized by the number 2, Figure 9C). It then continued to increase up to 4w and was followed by a decrease in the 3 RCCs (Figures 9A, C). This suggested a transient increase in RBC membrane microviscosity which was reminiscent to the transient rise of ATP concentration (Figures 3C, D). Accordingly, both parameters correlated positively (Figure 9D). Correlation with the EV number was also observed, but was negative between BODIPY-C10 lifetimes and the EV number at the same storage time. Correlation became positive if the lifetimes at 1 and 4w were correlated with EVs released at 4 and 7w respectively (Figures 9E, F). Altogether, these data suggested that the transient increase in membrane microviscosity preceded the release of EVs, at least in the 3 RCCs analysed. The release of EVs and chol from the RBC membrane could in turn affect the RBC membrane in such a way that other membrane regions could be affected.

## Discussion

### Main observations

**FIGURE 10:**
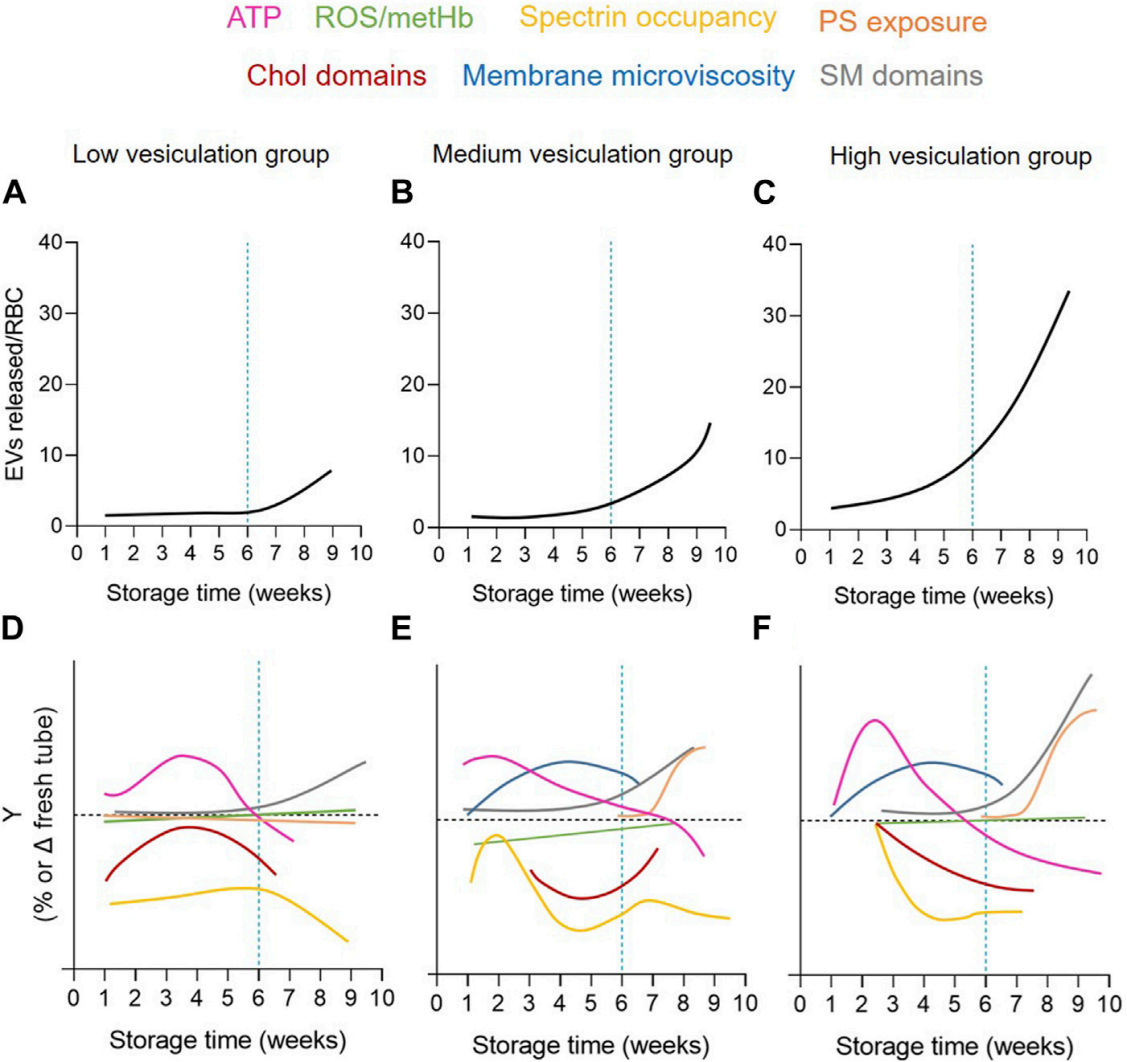
Graphical summary of the extent and kinetics of EV release and RBC alterations upon storage in the 3 vesiculation cohorts. The 3 RCC cohorts differed by the extent and kinetics of EV release **(A–C)** but also RBC membrane parameters, including (i) the cytoskeleton membrane occupancy (yellow) and chol-enriched domain abundance (red) during the 1-5w period; (ii) SM-enriched domain abundance (grey) from 5w; and (iii) PS-surface exposure (orange) from 8w **(D–F)**. On the other hand, the RBC membrane microviscosity (blue) and the intracellular ATP content (pink) evolved upon time but were not different between cohorts and ROS/metHb (green) did not appear modified.

The measurement of EV abundance revealed an exponential increase during storage but with a ∼40-fold variability between the 38 RCCs included in the study. These RCCs were subsequently classified into 3 cohorts based on their vesiculation rate (Figures 10A–C) and compared for intracellular and membrane parameters (Figures 10D–F). The variability in EV release at 6w was not associated with a differential ATP content or with increased oxidative stress (in the form of ROS, metHb and band3 integrity) but rather with RBC membrane modifications, i.e., cytoskeleton membrane occupancy, lateral heterogeneity in domains and transversal asymmetry, which were not affected simultaneously or with the same rate in each vesiculation group (Figures 10D–F). Among these membrane modifications, the decrease in chol-enriched domain abundance from the RBC surface suggested that those domains could represent a starting point for EV release and prompted us to decipher the mechanism.

### The RCCs exhibit an exponential release of EVs characterized by a constant size upon storage

The presence of vesicles in RCCs upon storage is no longer debated (Rubin et al., 2008; Prudent et al., 2018). However, their number and size vary largely between studies. As Rubin et al. and Lauren et al. (Rubin et al., 2008; Lauren et al., 2018), we showed that the EV number increased exponentially during storage. More than 2 × 10^7^ EVs/μL of RCC were measured at 42 days. This value is in concordance with Almizraq et al. who showed by tunable resistive pulse sensing that RCCs stored for 21 days contain between 0.5 and 2.5 × 10^7^ EVs/μL (Almizraq et al., 2017). Likewise, Lauren et al. reported by NTA that the number of EVs climbs up to ∼10^7^ EVs/μL after 42 days of storage (Lauren et al., 2018). Nevertheless, other groups reported considerably lower numbers, between 650 and 20,000 RBC-derived EVs/μL after 42 days (Rubin et al., 2008; Almizraq et al., 2017; Roussel et al., 2017; Gamonet et al., 2020). This difference could be related to the flow cytometry-based approaches which have difficulties to detect particles smaller than 200 nm (Serrano-Pertierra et al., 2020). Combined with the fact that optimal RBC markers (mostly GPA) are needed for these approaches, these studies have probably underestimated the number of EVs released in RCCs. Regarding EV size, most studies report an increase over storage (Kriebardis et al., 2008; Bicalho et al., 2016; Almizraq et al., 2017). In our study, the size of vesicles stayed stable all along storage. This difference might be related to different degrees of contamination. Indeed, Bicalho et al. and Almizraq et al. used differential centrifugation at low speed to isolate EVs whereas we observed that 2 steps of ultracentrifugation at 20,000 g were necessary to get rid of lipoprotein and platelet markers (Bicalho et al., 2016; Almizraq et al., 2017). Since both lipoproteins (except chylomicrons) and platelet-derived EVs are supposed to be smaller than EVs produced by RBCs, their presence could influence size measurements, especially at the beginning of storage during which RBC vesiculation is limited (Simonsen, 2017; Lopez et al., 2019).

### The RCCs can be classified into 3 cohorts based on the extent of EV release

The variability in the EV number between RCCs was previously reported and attributed to RCC processing, storage conditions, EV isolation and detection techniques as well as donor characteristics (Rubin et al., 2012; Bicalho et al., 2016; Almizraq et al., 2017; Almizraq et al., 2018; Gamonet et al., 2020; Serrano-Pertierra et al., 2020; Shopsowitz and Shih, 2021). We excluded a variability in RCC preparation as all RCCs were processed by La Croix-Rouge de Belgique. Contaminations and measurement irreproducibility due to EV isolation and detection methods were also dismissed as the comparison of EV kinetics upon storage of 4 RCCs originating from 2 same donors revealed reproducible results with NTA. Regarding donor characteristics, we cross-referenced the data for donor age, gender, BMI and blood group with the number of released EVs at 6w of storage for 25 RCCs. On this specific RCC cohort, no statistical difference could be detected for any of these donor characteristics (data not shown). Confusing data arise in the literature regarding this point. The study of Lelubre et al. showed that female and older donors are associated to increased RBC vesiculation tendency (Lelubre and Vincent, 2013) while the study of Gamonet et al., including 264 RCCs, showed no significant differences in EV abundance according to donor age or gender but described higher EV levels in RCCs from donors with the blood group B and with higher RBC counts (Gamonet et al., 2020). Moreover, in this global picture, the RBC parameters were generally not included, precluding the possibility to better understand the vesiculation mechanism. Here, by classification of RCCs into 3 cohorts based on their extent of vesiculation (Figures 10A–C) we were able to better understand the variability in EV release, both in quantity and kinetics.

### The variable EV number between cohorts does not result from differential metabolic impairments or from ROS and metHb accumulation but could be linked to distinct cytoskeleton occupancy alterations

In the medium and high vesiculation groups, the extent of spectrin membrane occupancy was close to fresh blood tubes at 3w of storage before abruptly decreasing. In contrast, in the low vesiculation cohort, this percentage was weaker than in fresh tubes from the beginning of storage and did not evolve until 6w (Figures 10D–F, yellow curves). The lower spectrin membrane occupancy is consistent with the study of Kozlova et al. who reported by atomic force microscopy the apparition of large pores formed in the RBC ghost cytoskeleton from 3w due to the rupture of cytoskeletal filaments (Kozlova et al., 2021; Sherstyukova et al., 2021). This alteration might be responsible for the rapid changes in RBC shape observed from 1w of storage. At this time point, echinocytes represented already more than 25% of RBCs. The abundance of echinocytes then continued to increase and even reached ∼60% at 6w.

One possible mechanism for the lower interaction between membrane and cytoskeleton is the ATP-dependent phosphorylation (Manno et al., 1995; Gov and Safran, 2005; Rinalducci et al., 2015). Indeed, Rinalducci et al. observed a substantial increase in the phosphorylation status at 3w of storage in RCCs and visualized by modelling that this event destabilizes interactions between ankyrin and β-spectrin (Rinalducci et al., 2015). In our study, ATP levels increased transiently up to 3w of storage before decreasing progressively. Despite the decrease, ATP levels remained elevated during the whole storage period in the 3 cohorts (Figures 10D–F, pink curves). As a consequence, exaggerated phosphorylation events could occur and alter the cytoskeleton occupancy. The transient increase of ATP agrees with Gevi et al. (Gevi et al., 2012) but is in discordance with other studies that described a slight but constant linear decrease (Burger et al., 2010; Karger et al., 2012; Livshits et al., 2021). During their *ex vivo* storage, RBCs are immersed in a highly concentrated glucose solution. Since the glucose transporter GLUT1 facilitates unidirectional glucose uptake along the concentration gradient, it is not surprising that RBCs react to their new environment and produce high ATP levels (Guizouarn and Allegrini, 2020). In favour of this hypothesis, we found that after 1w of storage the extracellular glucose concentration was ∼2-fold reduced, which implicated that half of the disposable glucose in SAGM (900 mg/dL) solution was used within this storage period (Burger et al., 2010). Additionally, it has been proposed that deoxygenated Hb binds to the cytoplasmic tail of band3 inducing the displacement of glycolytic enzymes allowing for their activation (Yoshida et al., 2019).

An alternative hypothesis for the impairment of membrane-cytoskeletal interactions is protein oxidation. Supporting this possibility, oxidative stress in RCCs has been described in the literature mainly in the form of protein and lipid oxidation or through the measurement of antioxidants such as glutathione and urate (D'Alessandro et al., 2012; Kriebardis et al., 2008; Gevi et al., 2012; Delobel et al., 2012). Although we measured variations in ROS content between RCCs, we did not observe any evolution over time (Supplementary Figure S8C; Figures 10D–F, green curves). Furthermore, the metHb content, a major target of ROS, was also not increased. This could be explained by the fact that ROS are extremely reactive and metHb unstable in hypothermic conditions (Shihana et al., 2011; Bardyn et al., 2018). We wondered therefore whether ROS could have attacked lipids or proteins other than Hb. The hypothesis that lipids could have been modified was rapidly discarded. Indeed, during the storage of K^+^/EDTA tubes, we previously reported that lipid peroxidation did not significantly increase during storage despite a strong and significant accumulation of ROS and metHb (Cloos et al., 2020). For this reason and since stored blood tubes are an accelerated model for vesiculation, we excluded the possibility that lipid peroxidation would be consequential in stored RCCs. In addition to lipids and Hb, band3, a key component of the membrane-cytoskeleton anchorage complexes, is a preferential target for oxidative attack that leads to its aggregation in membrane and loss in EVs during storage (Prudent et al., 2018). As several research groups, we detected the presence of band3 in EVs from 3w as well as degradation products in RBC ghosts after 3w of storage, suggesting a certain level of oxidative damage at least at the protein level (Rinalducci et al., 2012; Prudent et al., 2018; Freitas Leal et al., 2020). However, band3 dimers in EVs and band3 fragments in RBC ghosts did not accumulate upon storage and showed no obvious differences between the 3 vesiculation cohorts.

### The differential EV release in the 3 cohorts is associated with unequal and late impairments of membrane transversal asymmetry during storage

In contrast to the cytoskeleton occupancy, the RBC transversal asymmetry was maintained during the whole legal storage period and started to be altered only after 8w, to reach ∼3% of PS-exposing cells in the highest vesiculation group (Figures 10D–F, orange curves). This is in agreement with previous studies which have also reported a limited increase in PS exposure with storage time with maximum levels seen at 7w (Verhoeven et al., 2006; Burger et al., 2010; Dinkla et al., 2014). This very late and limited increase could result from the early loss of PS by vesiculation. Indeed, Freitas Leal et al. observed that nearly 100% of EVs were PS-positive after 3w (Freitas Leal et al., 2020). Alternatively, the use of fluorescent Annexin-V could have underestimated the number of PS-exposing RBCs since Lu et al. detected 18% of PS-exposed RBCs with lactadherin at 6w of storage in RCCs *versus* only 4.5% with Annexin-V (Lu et al., 2011). Nevertheless, low PS exposure in the present storage conditions should be expected and might be explained by the independency of PS exposure from enzymes implicated in transversal asymmetry regulation. In fact, the transversal asymmetry could be largely preserved as calcium is absent in the SAGM solution, preventing the activation of scramblase, and as ATP stores are maintained high until 6w, allowing for flippase activity. Besides calcium, the RBC membrane chol content is also involved in the control of transversal asymmetry and could play a role here. Indeed, in our previous study on RBC storage in K^+^/EDTA-coated tubes, we observed that the restoration of the RBC chol content is associated to restored levels of PS exposing RBCs (Cloos et al., 2020). In the present study, we revealed that the highest vesiculation group showed a clear decrease of the RBC chol content, as described for stored RBCs in blood tubes, and the highest increase in PS exposure, which supports this last hypothesis. Nevertheless, even if the mechanism behind PS exposure remains to be elucidated, this alteration was late in all vesiculation groups, reducing the possibility that it could represent a triggering factor for early EV production.

### The variability in EV release in the 3 cohorts is associated with a late and variable increase of SM-enriched domains

The 3 vesiculation cohorts can also be distinguished based on the abundance of SM-enriched domains. The increase in SM-domains abundance was consistent with the maintenance of SM species in stored RBCs and the similar SM enrichment of EVs compared with their parent cells (Lauren et al., 2018; Freitas Leal et al., 2020). Among the triggering factors for SM-enriched domain increase, we can consider the impairment of membrane-cytoskeleton interaction. Indeed, the spectrin membrane occupancy started to decrease at ∼6w, 2w and 3w, in the 3 cohorts respectively while the increase in SM-enriched domains started to rise with a delay of 2–3w (Figures 10D–F, grey curves). Additionally, the decrease in chol-enriched domain abundance might represent an another or additional cause for SM-enriched domain alterations, resulting from impairment of RBC global or local membrane fluidity and/or curvature, both regulated by chol (Leonard et al., 2017a).

### The differential EV release in the 3 cohorts is associated with a decrease of chol-enriched domains at different times of storage

The chol-enriched domain decrease started from 2w of storage and was specifically observed in RBC HC areas. We therefore asked whether these domains could represent the starting point for EV departure from specific regions of the RBC surface upon storage. Four lines of evidence support this hypothesis. First, the abundance of chol-enriched domains at the HC areas correlated with EV release, at least in the low and medium vesiculation groups (data not shown). Second, as previously shown (Salzer et al., 2008; Freitas Leal et al., 2020), the chol-binding protein stomatin was more enriched in EVs compared with RBC ghosts and with the raft protein flotillin-1 at 6w. This is interesting since stomatin displays a scaffolding function by assembling small membrane microdomains to form larger complexes and to regulate the activity of membrane proteins (Salzer et al., 2007). Third, chol was found in EVs from the 3 cohorts but was more abundant in the high vesiculation group. Finally, the reduction in the abundance of chol-enriched domains was also observed in stored blood tubes in contrast to SM-enriched domains (Cloos et al., 2020). Altogether, our data support the hypothesis that the decrease of chol-enriched domains at the RBC surface did not result from a redistribution of chol in the bilayer but rather from their release by vesiculation.

On a mechanistic point-of-view, based on our previous study on K^+^/EDTA tubes upon storage at 4 °C, the increase in membrane microviscosity (Figures 10E,F, blue curves) during storage could represent the starting point for chol-enriched domain loss from the RBC surface. Nevertheless, the opposite hypothesis that loss of chol-enriched domains impacts membrane viscosity cannot be excluded. Such increase in membrane microviscosity was already shown by Kozlova et al. from 4w of storage (Kozlova et al., 2021). The mechanism behind is not clear. Some studies have suggested that it could result from the ATP decrease (Xu et al., 2018). Others rather attributed the decrease to the rupture of cytoskeletal proteins dissociations with the bilayer by phosphorylation and oxidation, leading to the aggregation of membrane components and thickening of cytoskeletal filaments. As a consequence, the membrane organisation and composition change and the cytoskeleton rearrange abnormally with the RBC surface triggering new tensions and an increase in the membrane microviscosity (Kozlova et al., 2021). Additionally, the hypothermic storage could also be implicated as the lower temperature decreases the membrane fluidity (Nozawa, 2011) and the activity of glycolytic and antioxidant enzymes during storage (Yoshida et al., 2019). As a result, lipid peroxidation has been demonstrated through detection of malondialdehyde (D'Alessandro et al., 2012; Bardyn et al., 2018; Collard et al., 2014; Dumaswala et al., 1999), changing membrane composition and organisation. Whatever the mechanism, the increase in microviscosity could cause a line tension at the edges of chol-enriched domains, inducing their release by vesiculation, as previously suggested (Leonard et al., 2017b; Leonard et al., 2018).

Nevertheless, chol-enriched domains started to re-increase from 5w. As this perfectly coincided with the beginning of SM-enriched domain increase in the LC areas and as SM-enriched domains are coenriched in chol (Conrard et al., 2018), one can reasonably propose that the re-increase of chol-enriched domains reflects the increase of SM-enriched domains observed in LC areas from 5w. However, this is probably not the only explanation since chol-enriched domains also re-increased in HC areas. This observation could result from the fact that, after losing chol, the HC area becomes more fluid, facilitating lipid lateral diffusion and allowing new domains to be recruited or to newly form. Those domains could contain polar lipids (e.g., SM or GM1 ganglioside) as well and be lost by vesiculation at later time point, providing a potential explanation behind the positive correlation between the kinetics of chol-enriched domains and EV chol content upon storage in the high vesiculation group. This possibility is supported by the fact that the RBC membrane chol content also specifically decreased in this group upon storage. These data suggested that the decrease of chol-enriched domains represented probably only a part of the global vesiculation mechanism in the high vesiculation group.

### Study limitations

Although this study included 38 RCCs from 36 different donors, it encountered limitations. First, most of RCCs were not followed during the whole storage period, meaning that several time intervals did not comprise the exact same RCCs and potentially introducing donor-related issues. However, we did not detect statistical differences for 25 RCCs regarding the following donor characteristics: age, gender, BMI and blood groups. Second, since a large number of RCCs were not studied from day 0, early time points for the different parameters require to be further investigated. This is not easy to implement since RCCs are intended to be transfused at those times. To overcome this difficulty, data for most parameters were expressed in relation to values obtained on fresh whole blood tubes from the same control donor, avoiding data misinterpretation due to experimental variability. To ensure this internal control did not alter the real kinetics of each RCC, we followed 2 RCCs throughout the whole storage period for the main parameters (i.e., EV abundance, ATP and ROS contents, PS exposure, chol-enriched domain abundance and membrane chol content) and revealed that, whatever the parameter, the 2 RCCs followed the trend of the overall cohort (Supplementary Figure S8). Third, by stratifying our RCC cohort into 3 groups and working with weekly storage intervals, we reduced the number of RCCs studied which complicated the statistical analysis and impacted the probability to reach statistical significance. Nevertheless, despite the absence of statistical analysis or the lack of statistical significance for some parameters, we commented on the observed alterations to encourage the scientific community to further characterize those showing differences between cohorts. Finally, although studying the 8–10w period overtook the acceptable *in vitro* status, it allowed us to confirm that the 3 vesiculation cohorts behave differently, to better understand how and when lesions became substantial and to pave the way for extending the storage time of the low vesiculation cohort.

## Conclusion

Our data reveal for the first time that the differential extent of EV release in RCCs did not simply result from RCC preparation method, storage conditions or technical EV-related issues but could be linked to RBC membrane alterations, which are distinct in the 3 cohorts both in terms of extent and kinetics. Among those, the cytoskeleton occupancy changes and the kinetics of SM-enriched domain rise and of PS exposure increase could be distinguished between the 3 groups. Moreover, EV release from specific chol-enriched domains occurs in the 3 vesiculation cohorts but at different time points and they show differential correlation with the EV chol content. These results implicate that chol-enriched domains, combined or not with membrane microviscosity, could represent a potential target to limit EV generation in RCCs since they represent two early membrane alterations. However, in the light of the protective role of EVs in RBC survival and functionality, the contribution of lipid domains in membrane signaling and the significant role of chol in membrane homeostasis, a better understanding of the relationship between those two parameters as well as the precise kinetics in the 3 cohorts is absolutely required.

## Data Availability

The original contributions presented in the study are included in the article/Supplementary Material, further inquiries can be directed to the corresponding author.
