# Corrigendum: Variability of extracellular vesicle release during storage of red blood cell concentrates is associated with differential membrane alterations, including loss of cholesterol-enriched domains

**DOI:** 10.3389/fphys.2023.1291218

**Published:** 2023-09-18

**Authors:** Marine Ghodsi, Anne-Sophie Cloos, Negar Mozaheb, Patrick Van Der Smissen, Patrick Henriet, Christophe E. Pierreux, Nicolas Cellier, Marie-Paule Mingeot-Leclercq, Tomé Najdovski, Donatienne Tyteca

**Affiliations:** ^1^ Cell Biology Unit and Platform for Imaging Cells and Tissues, de Duve Institute, UCLouvain, Brussels, Belgium; ^2^ Cellular and Molecular Pharmacology Unit, Louvain Drug Research Institute, UCLouvain, Brussels, Belgium; ^3^ Service du Sang, Croix-Rouge de Belgique, Suarlée, Belgium

**Keywords:** red blood cell transfusion, intracellular ATP, oxidative stress, spectrin network, cholesterol, phosphatidylserine surface exposure, sphingomyelin-enriched domains, membrane microviscosity

In the published article, an **Author name** was incorrectly written as Marie-Paule Mingeot. The correct spelling is Marie-Paule Mingeot-Leclercq.

The authors apologize for this error and state that this does not change the scientific conclusions of the article in any way. The original article has been updated.

