# Supplementary figures and images for: Variability of extracellular vesicle release during storage of red blood cell concentrates is associated with differential membrane alterations, including loss of cholesterol-enriched domains
Source: Front Physiol. 2023 Jun 20;14:1205493. doi: 10.3389/fphys.2023.1205493 (PMC10318158; doi:10.3389/fphys.2023.1205493)

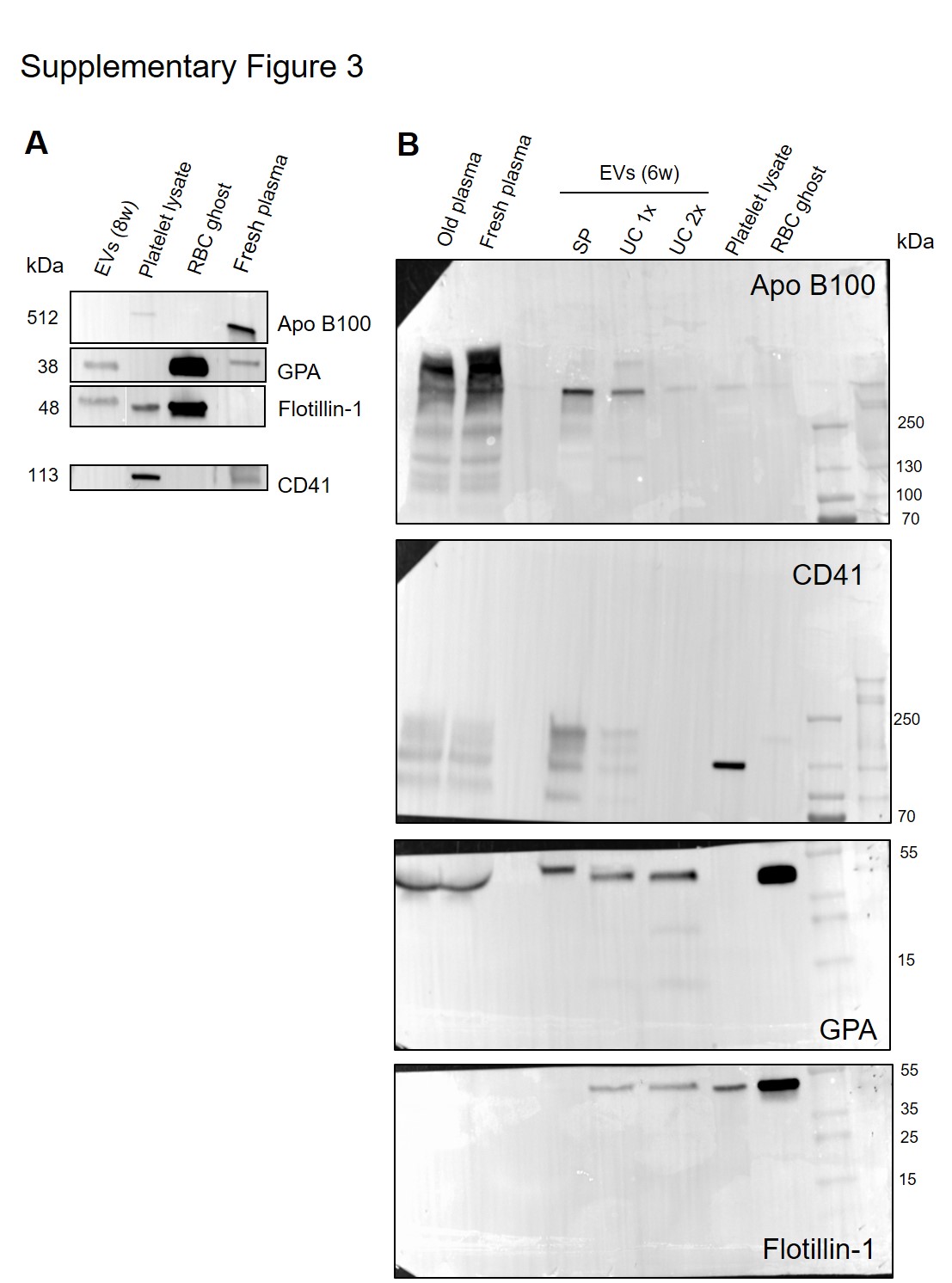

Supplement: Supplementary file 1 [file Image3.JPEG]

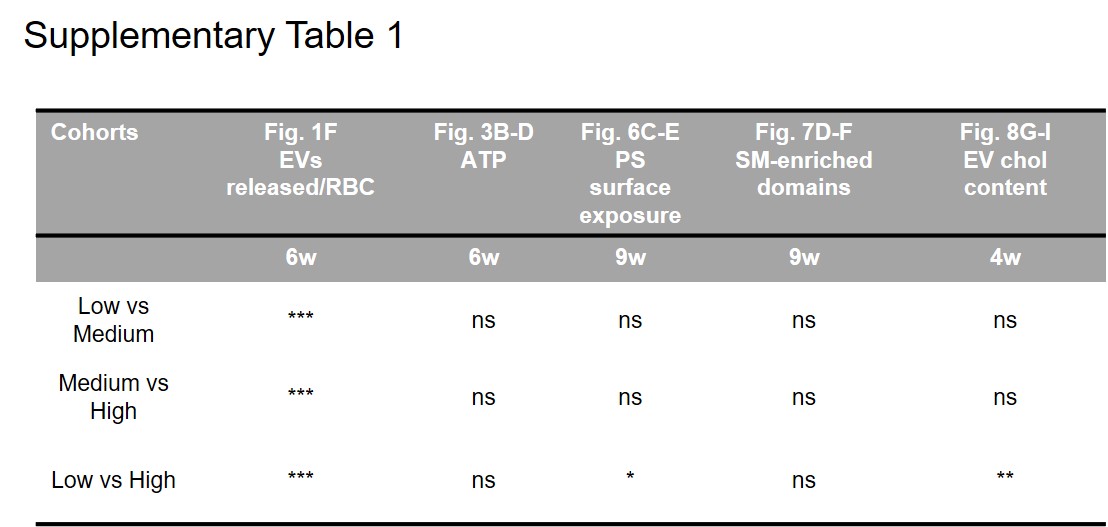

Supplement: Supplementary file 3 [file Image9.JPEG]

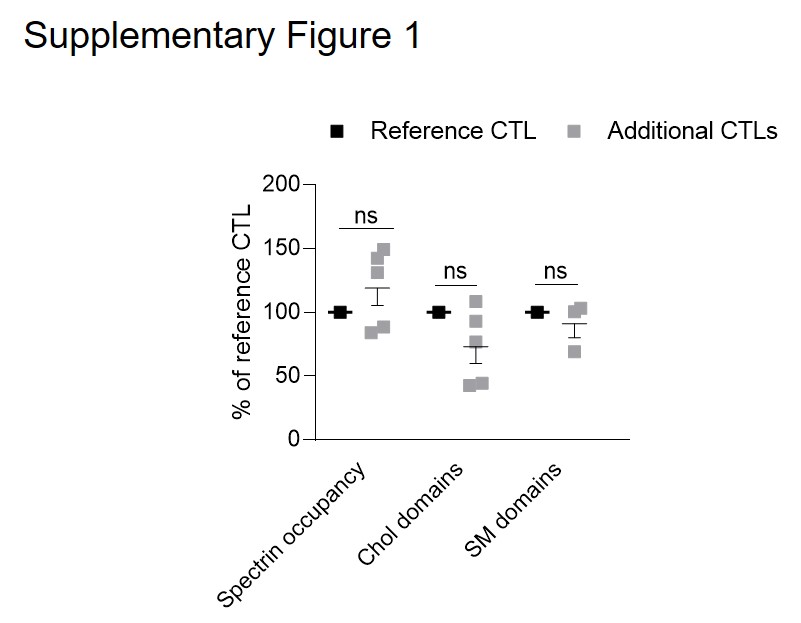

Supplement: Supplementary file 5 [file Image1.JPEG]

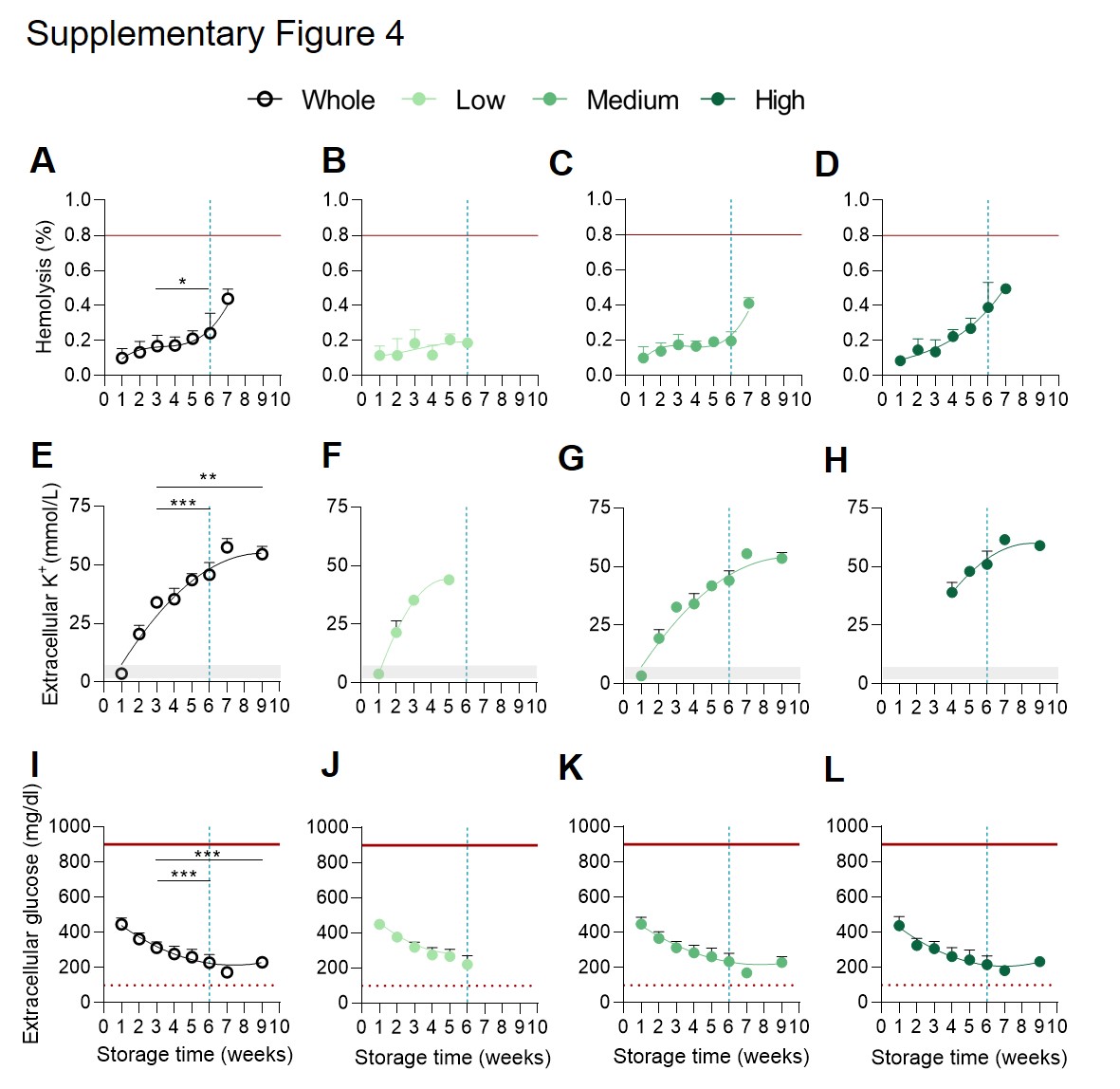

Supplement: Supplementary file 6 [file Image4.JPEG]

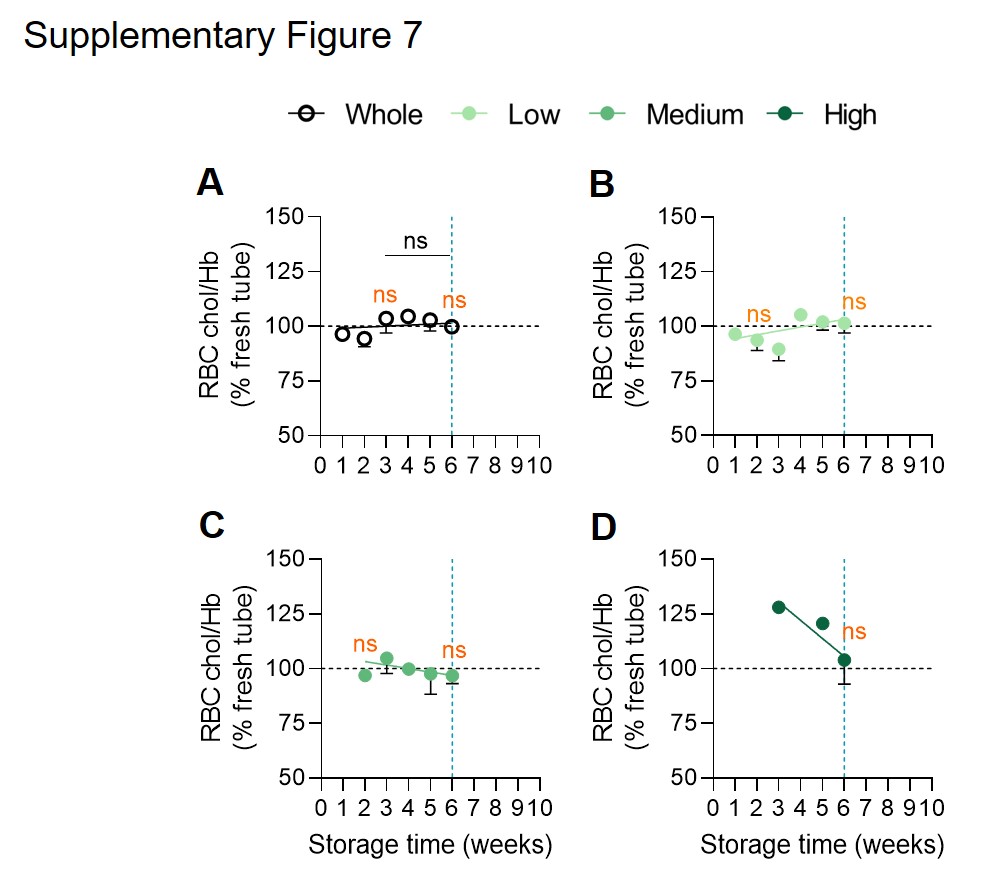

Supplement: Supplementary file 7 [file Image7.JPEG]

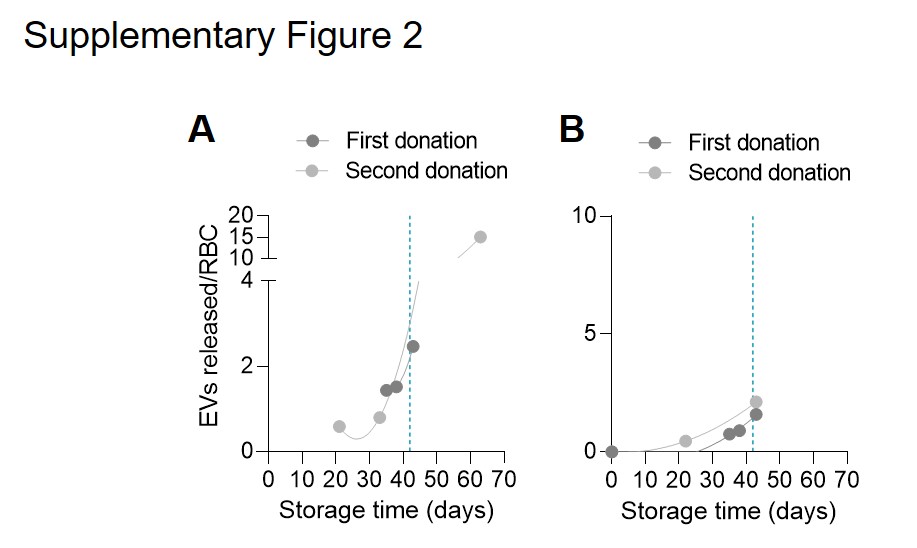

Supplement: Supplementary file 8 [file Image2.JPEG]

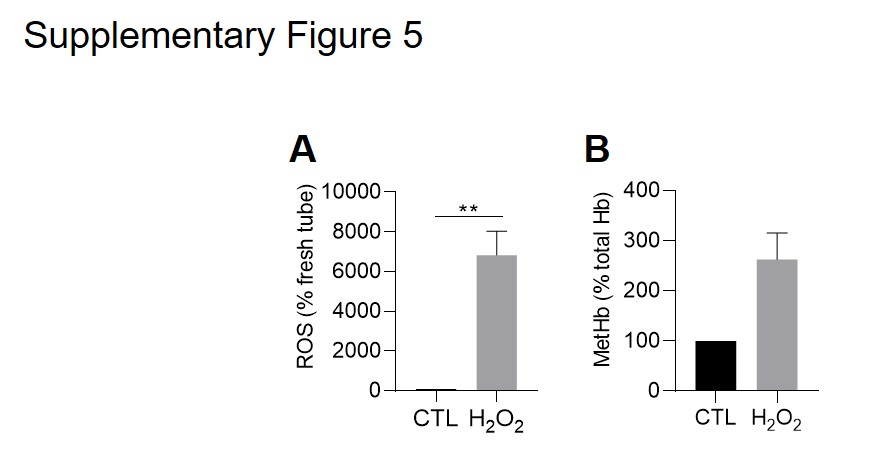

Supplement: Supplementary file 9 [file Image5.JPEG]

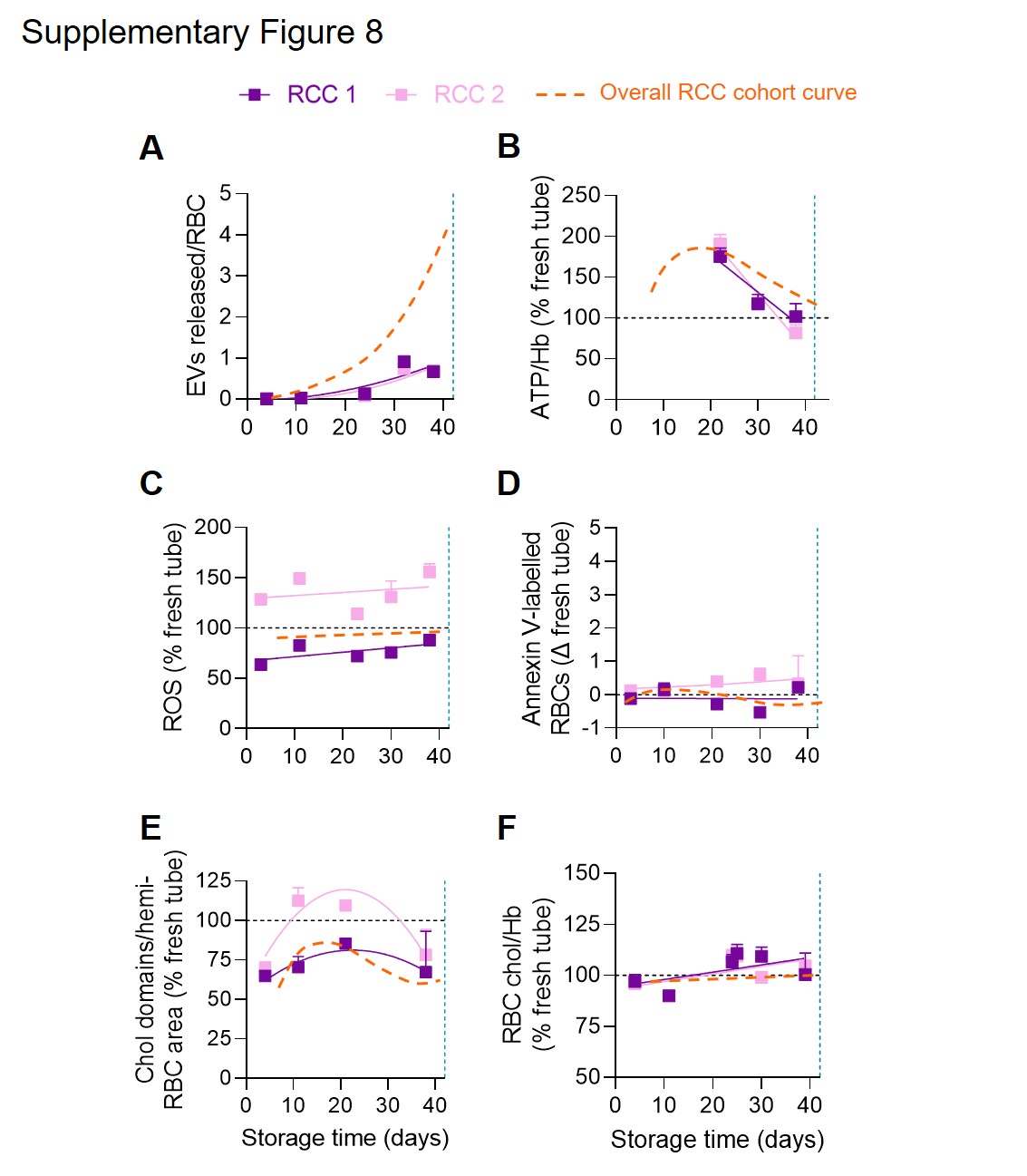

Supplement: Supplementary file 10 [file Image8.JPEG]

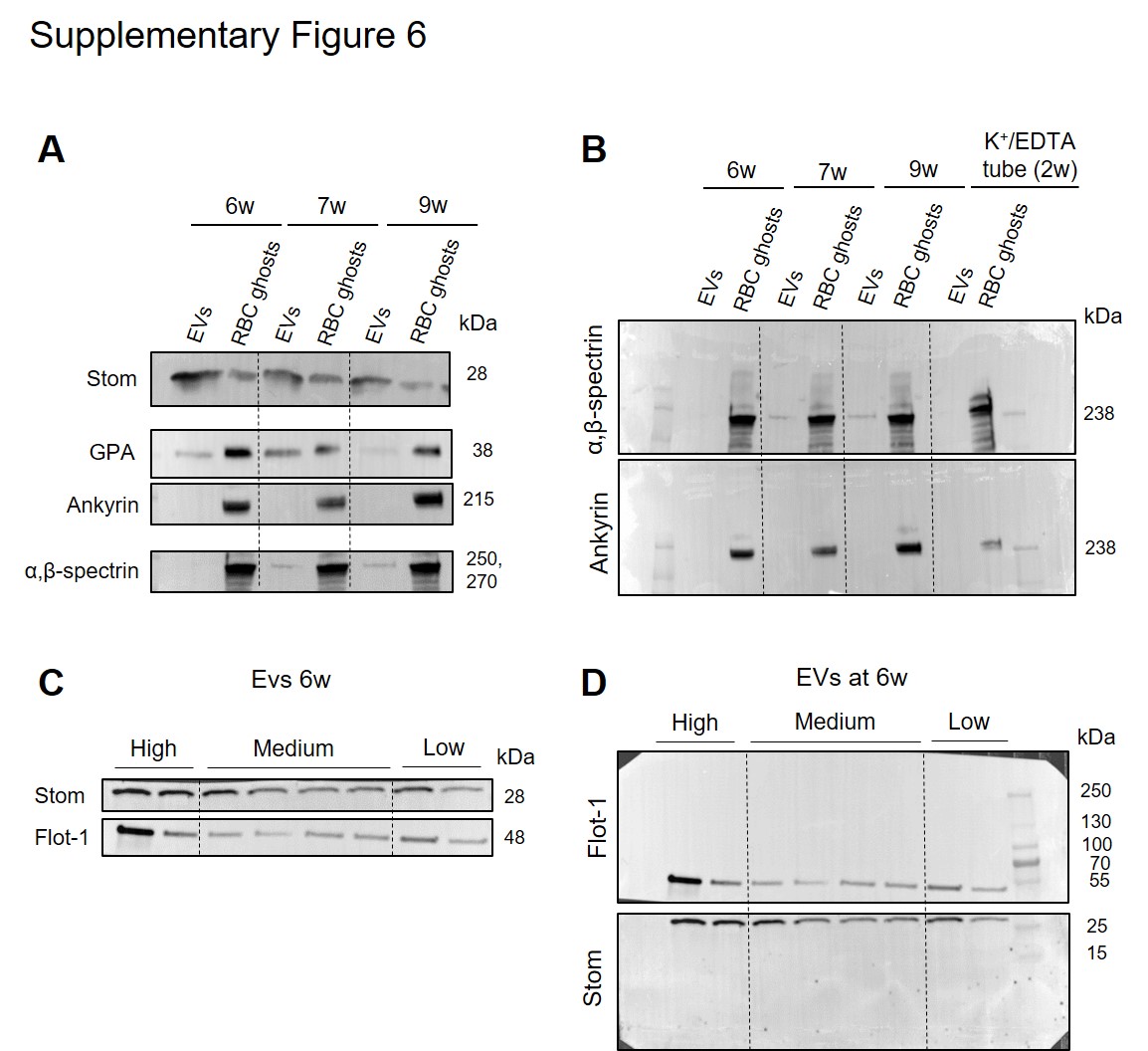

Supplement: Supplementary file 11 [file Image6.JPEG]
