# Supplementary material for: Variability of extracellular vesicle release during storage of red blood cell concentrates is associated with differential membrane alterations, including loss of cholesterol-enriched domains
Source: Front Physiol. 2023 Jun 20;14:1205493. doi: 10.3389/fphys.2023.1205493 (PMC10318158; doi:10.3389/fphys.2023.1205493)
Supplement: Supplementary file 2 [file Table1.pdf]

Supplementary Table 1

Cohorts	Fig. 1F EVs released/RBC	Fig. 3B-D ATP	Fig. 6C-E PS surface exposure	Fig. 7D-F SM-enriched domains	Fig. 8G-I EV chol content
	6w	6w	9w	9w	3w
Low vs Medium	***	ns	ns	ns	ns
Medium vs High	***	ns	ns	ns	ns
Low vs High	***	ns	*	ns	**
