# Supplementary material for: Variability of extracellular vesicle release during storage of red blood cell concentrates is associated with differential membrane alterations, including loss of cholesterol-enriched domains
Source: Front Physiol. 2023 Jun 20;14:1205493. doi: 10.3389/fphys.2023.1205493 (PMC10318158; doi:10.3389/fphys.2023.1205493)
Supplement: Supplementary file 4 [file Presentation1.pdf]

Variability of extracellular vesicle release during storage of red blood cell concentrates is associated with differential membrane alterations, including loss of cholesterol-enriched domains

Marine Ghodsi[1*], Anne-Sophie Cloos[1*], Negar Mozaheb[2], Patrick Van Der Smissen[1], Patrick Henriet[1], Christophe E. Pierreux[1], Nicolas Cellier[3], Marie-Paule Mingeot-Leclercq[2], Tomé Najdovski[3] and Donatienne Tyteca[1]

[1] Cell biology Unit & Platform for Imaging Cells and Tissues, de Duve Institute, UCLouvain, Brussels, Belgium

[2] Cellular and molecular pharmacology Unit, Louvain Drug Research Institute, UCLouvain, Brussels, Belgium

[3] Service du Sang, Croix-Rouge de Belgique, Suarlée, Belgium

* Contributed equally

Corresponding author: Donatienne Tyteca, Cell biology Unit & Platform for Imaging Cells and Tissues, de Duve Institute, UCLouvain, UCL B1.75.05, Avenue Hippocrate, 75, B-1200 Brussels, Belgium. Phone: +32-2-764.75.91; Fax: +32-2-764.75.43; e-mail: donatienne.tyteca@uclouvain.be

Keywords: red blood cell transfusion, intracellular ATP, oxidative stress, spectrin network, cholesterol, phosphatidylserine surface exposure, sphingomyelin-enriched domains, membrane microviscosity

Supplemental figure legends

Supplementary Table 1: Statistical comparison between the 3 vesiculation cohorts, at a specific time point of storage for different parameters. One-way ANOVA followed by Tukey's post-hoc test or Kruskall-Wallis test followed by Dunn's multiple comparisons.

Supplementary Figure 1: The RBC membrane parameters of the reference blood tube donor used in the study do not significantly differ from additional donors. The female reference blood tube donor included in the study as internal control (black) was compared with 3-5 additional donors (grey) for spectrin-membrane occupancy and abundance of chol- and SM-enriched domains. These parameters were evaluated to ensure that the reference control does not differ and interfere with data analysis of RCCs. Data are expressed as mean ± SEM. Wilcoxon signed rank test.

Supplementary Figure 2: The kinetics of EV release between 2 RCC donations obtained from the same donor are similar. A, Comparison of the vesiculation kinetics of two RCCs provided at 1-year interval by the same donor and included in the medium vesiculation cohort. **B,** Comparison of the vesiculation kinetics of two RCCs provided at 2-years interval by the same donor and included in the low vesiculation group.

Supplementary Figure 3: The particle preparation is not contaminated by lipoproteins or platelets (or their EVs) and contains the raft marker flotillin-1 (extension of Fig. 1C). A, The experiment of **Figure 1C** was reproduced with a second RCC at 8w of storage. 20 µg/well of particles and RBC ghost proteins. Apo B100 and GPA were revealed on the same cut membrane. Flotillin-1 was revealed after GPA membrane stripping. CD41 was revealed on a second membrane. **B,** Representative Western blots. Purity of particle preparations was evaluated by Western blotting at each step of the differential centrifugation protocol for lipoprotein (Apo B100) and platelet (CD41) contaminations as well as for the presence of RBC (GPA) and EV (flotillin-1) markers. Fresh plasma, plasma from blood tubes stored for 5 days, platelet lysates and RBC ghosts were used as positive controls. All markers were detected in the supernatant (SP) collected after the low speed centrifugation to separate RBCs from SAGM medium, in the pellet recovered after the first ultracentrifugation step (UC1) or the second ultracentrifugation step (UC2). 20 µg/well of particles and RBC ghost proteins.

Supplementary Figure 4: Hemolysis and extracellular potassium and glucose concentration do not differ between the 3 vesiculation cohorts. (A-D) Hemolysis. Evolution upon time in the overall RCC population (**A**, n=14 RCCs) or in each vesiculation cohort (**B**, n=3; **C**, n=8; **D**, n=3 RCCs) of percentage of hemolysis calculated as follows: (100-hematocrit) x supernatant Hb/total Hb. Parameters were determined with the automated blood gas analyser. The legal level allowed by the Council of Europe

guidelines of 0.8% at 6w is indicated by the red lines at the top of the graphs. Data are expressed as mean ± SD. Unpaired t test after logarithmic transformation. **(E-H) Extracellular potassium.** Evolution upon time in the overall RCC population (**E**, n=10 RCCs) or in each vesiculation cohort (**F**, n=1; **G**, n=7; **H**, n=2 RCCs) of K^+ levels determined with the automated blood gas analyser in supernatants separated from RCCs through centrifugation at low speed. The physiological range of plasma K^+ levels is represented by the clear grey frame. Data are expressed as mean ± SD. Mann-Whitney t test. **(I-L) Extracellular glucose**. Evolution upon time of glucose concentration in the overall RCC population (**I**, n=22 RCCs) or in each vesiculation cohort (**J**, n=5; **K**, n=13; **L**, n=4 RCCs). The glucose concentration in SAGM (900 mg/dl) and plasma (100 mg/dl) is indicated by the red lines at the top and bottom of the graphs. Data are expressed as mean ± SD. Mann-Whitney t test. Statistical test is indicated above a line connecting 2 time intervals.

Supplementary Figure 5: ROS and metHb accumulate in fresh blood tubes after H_2O_2 treatment. A, ROS accumulation. RBCs from fresh blood tubes were treated with H_2O_2 and used as positive control. Data are expressed as mean ± SEM (n=7). One sample t test. **B, MetHb accumulation**. RBCs from fresh blood tubes were treated with H_2O_2 and used as positive control. Data are expressed as mean ± SD (n=2).

Supplementary Figure 6: The chol-binding protein stomatin is enriched in EVs as compared with membrane proteins, glycophorin A and flotillin-1, while cytoskeletal proteins are almost absent. A,B, Western blotting of stomatin in ghosts and EVs of RCCs at the indicated times by comparison with other membrane and cytoskeletal proteins (GPA, ankyrin, spectrin). 16.5 µg/well of EVs and RBC ghost proteins for all storage time. GPA and ankyrin were revealed on the same cut membrane. Stomatin and spectrin were revealed independently on two other membranes. In **B**, EVs and ghosts from stored K^+/EDTA tubes were added as internal controls (9.5 µg/well of EV and RBC ghost proteins). These blots are representative of 3 RCCs. **C,D**, Western blotting of stomatin in EVs at 6w in each vesiculation cohort compared with the raft marker flotillin-1 (n=8 RCCs). 2.2 µg/well of EVs proteins. Stomatin and flotillin-1 were revealed on the same cut membrane.

Supplementary Figure 7: The RBC membrane cholesterol content remains stable upon storage in the low and medium cohorts whereas it tends to decrease in the high vesiculation group. Chol content was determined as in **Figures 8G-I** except that data were normalised by the Hb content and expressed as % of fresh blood tubes (horizontal black dotted lines). Evolution of chol enrichment in RBCs upon time in the overall population (**A**, n= 18 RCCs) and in the 3 groups of vesiculation (**B**, n=6; **C**, n=8; **D**, n=4 RCCs). Data are expressed as mean ± SEM. Unpaired t test (for the entire cohort) or Mann-Whitney test (for each cohort individually). Statistical significance is indicated above a line connecting 2 time

intervals. In orange, one sample t test (for the entire cohort) or Wilcoxon signed rank test (for each cohort individually) are represented above a precise time interval to give comparison with the internal control (fresh blood tube).

Supplementary Figure 8: The evolution of 6 biochemical and membrane parameters on 2 independent RCCs upon storage does not differ from the trends observed in the overall RCC cohort. Two RCCs (represented in purple and pink) from the low vesiculation cohort were followed during the whole storage period and studied for 6 parameters: **A**, EV abundance; **B**, ATP content; **C**, ROS content; **D**, PS exposure; **E**, chol-enriched domain abundance; and **F**, membrane chol content. The evolution curves from the overall RCC cohort for the 6 parameters were represented in orange dotted lines.
